# Investigation of a mouse model of Prader-Willi Syndrome with combined disruption of *Necdin* and *Magel2*

**DOI:** 10.1172/jci.insight.185159

**Published:** 2025-03-06

**Authors:** Pierre-Yves Barelle, Alicia Sicardi, Fabienne Schaller, Julie Buron, Denis Becquet, Felix Omnes, Françoise Watrin, Marie-Sophie Alifrangis, Catarina Santos, Clément Menuet, Anne-Marie François-Bellan, Emilie Caron, Jessica Klucznik, Vincent Prevot, Sebastien G. Bouret, Françoise Muscatelli

**Affiliations:** 1 University Lille, Inserm, CHU Lille, Laboratory of Development and Plasticity of the Neuroendocrine Brain, Lille Neuroscience and Cognition, UMR-S 1172, Lille F-59000, France.; 2 FHU 1000 Days for Health, School of Medicine, Lille F-59000, France.; 3 Institut de Neurobiologie de la Méditerranée (INMED), INSERM, Aix Marseille Université, Marseille, France.; 4 University of Aix-Marseille, Inst Neurophysiopathol, Marseille, France.; 5 Phenotype Expertise, Marseille, France.

**Keywords:** Genetics, Neuroscience, Genetic diseases, Mouse models, Neuroendocrine regulation

## Abstract

Prader-Willi syndrome (PWS) is a multigenic disorder caused by the loss of 7 contiguous paternally expressed genes. Mouse models with inactivation of all PWS genes are lethal. KO mouse models for each candidate gene have been generated, but they lack the functional interactions between PWS genes. Here, we revealed an interplay between *Necdin* and *Magel2* PWS genes and generated a mouse model (named *Del Ndn-Magel2* mice) with a deletion including both genes. A subset of *Del Ndn-Magel2* mice showed neonatal lethality. Behaviorally, surviving mutant mice exhibited sensory delays during infancy and alterations in social exploration at adulthood. *Del Ndn-Magel2* mice had a lower body weight before weaning, persisting after weaning in males only, with reduced fat mass and improved glucose tolerance as well as altered puberty. Adult mutant mice displayed increased ventilation and a persistent increase in apneas following a hypercapnic challenge. Transcriptomics analyses revealed a dysregulation of key circadian genes and alterations of genes associated with axonal function similar to patients with PWS. At neuroanatomical levels, *Del Ndn-Magel2* mice had an impaired maturation of oxytocin neurons and a disrupted development of melanocortin circuits. Together, these data indicate that the *Del Ndn-Magel2* mouse is a pertinent and genetically relevant model of PWS.

## Abstract 2

A mouse model of PWS with Necdin and Magel2 deletion showed neonatal lethality, behavioral, metabolic, and neuroanatomical alterations that closely parallel clinical features of PWS

## Graphical abstract

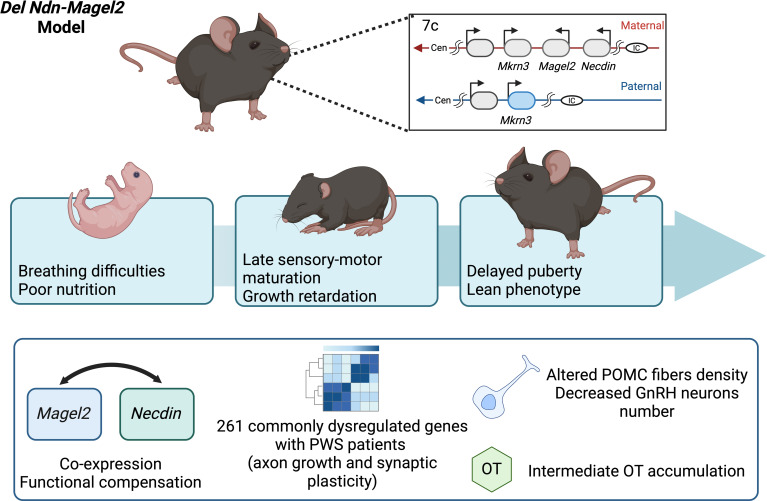

## Introduction

Prader-Willi syndrome (PWS) arises from the loss of expression of 7 contiguous paternally inherited genes located in the 15q11-q13 region, including the *MAGEL2* and *NECDIN* genes. All the PWS genes are regulated by genomic imprinting, which is an epigenetic process where only 1 allele is expressed in a parent-of-origin–dependent manner. PWS is a complex neurodevelopmental disorder characterized by a lifelong spectrum of phenotypic features starting with severe feeding difficulties and respiratory distress at birth, early sensory deficits, and hypotonia, followed by growth deficiency, hypogonadism and delayed puberty, short stature, excessive weight gain with severe hyperphagia, and cognitive and behavioral problems throughout life (1, 2). Despite extensive clinical trials, there are no effective treatments for PWS, and comprehensive pathophysiological mechanisms have not yet been clearly identified, although converging evidence suggests that the PWS phenotype might result from hypothalamic dysfunctions (2, 3). The contribution of candidate genes in the pathogenesis of PWS and how they interact between each other is still under debate. Nevertheless, 7 rare patients with PWS have a chromosomal deletion including *SNORD116* but not *SNORD115* (noncoding small nucleolar RNA C/D clusters [SNORDs]) (4), suggesting an important role of *SNORD116*. However, 2 others patients have a normal expression of *SNORD116* yet display a PWS-like phenotype (5, 6). In addition, point mutations in the paternal allele of *MAGEL2* only are responsible for the Schaaf-Yang syndrome (SYS) that has a phenotypic overlap with PWS but with a more severe autistic phenotype in adolescence and adulthood (7, 8). These results suggest that the loss of function of *MAGEL2* is responsible for overlapping symptoms in PWS and SYS.

Animal models for PWS are instrumental in understanding mechanisms and identifying novel pathways involved in the pathophysiology of PWS. The mouse chromosome 7C presents a conserved synteny to the human PWS region. However, mouse models with inactivation of all PWS genes display a 100% lethality rate within the first week after birth and have, therefore, not been very useful for understanding postnatal symptoms (9, 10). Mouse KO models for single candidate genes have also been generated (11). However, these single KO models have limitations, since it is likely that the PWS phenotype is the result of the lack of expression of several genes that are coexpressed in the same brain regions (12) and that may interact with each other, creating a more complex and integrative phenotype.

Among the genes inactivated in PWS, SNORDs *NECDIN* and *MAGEL2* are of particular interest. *Snord116-*KO mice display some lethality before weaning, and they display growth delay but consume proportionally more food, considered as hyperphagia (13–15), although they do not become obese (13, 14). They also exhibit cognitive deficits (16) and sleep behavior alterations (17). However, *Snord115*-KO mice appear normal with no obvious behavioral or metabolic alterations (18, 19). *Necdin* and *Magel2*–single KO mouse models are also of particular interest since they display several distinct phenotypes mimicking part of the PWS clinical features, although there is variability among the different models depending on the genomic construction. *Magel2*-KO mice exhibit suckling deficits at birth (20), growth retardation (20), altered metabolism (21, 22), circadian activity disturbances (23), and deficits in cognitive, social, and parental behaviors (24–27). Our teams and others also reported impaired hypothalamic regulation in *Magel2*-KO mice with abnormal oxytocin (OT) maturation (20, 24, 28, 29) and disrupted development and function of proopiomelanocortin (POMC) neurons (29, 30). *Necdin*-KO mice display variable lethality after birth (31, 32) due to respiratory distress (33), growth retardation, motor deficit in infancy (34), sensory deficits (35), high scraping, cognitive alterations (32), and alterations of social and circadian behaviors (36, 37). At the neuroanatomical level, *Necdin*-KO mice display a reduction in the number of OT and gonadotropin-releasing hormone–producing (GnRH-producing) neurons, alterations in perinatal serotonergic metabolism and development (32, 38), and alterations in clock gene expression (36).

*MAGEL2* and *NECDIN* belong to the melanoma antigen gene expression (MAGE) gene family (39). They are physically close in the genome (30 kb), are without introns, and have probably evolved through sequential retrotransposition events (12). In addition, they are coexpressed in many brain structures, including in the developing hypothalamus (12, 40, 41). At the molecular level, both proteins act through a ubiquitin-dependent mechanism to turn over and recycle proteins (42). Overall, their molecular function and expression pattern suggest that their roles in cellular processes may partially overlap (42). Therefore, an animal model with the combined loss of *Magel2* and *Necdin* versus a single invalidation of each gene should reveal the complex interaction of these 2 genes and avoid the potential functional redundancy or compensatory mechanism between them. This model would therefore be more relevant, compared with single KO mice, to study the complexity of PWS.

In the present study, we investigated the interplay between *Necdin* and *Magel2* genes, generated a mouse model with a deletion including both *Magel2* and *Necdin* genes, and provided a comprehensive characterization of the behavioral, physiological, neurodevelopmental, and transcriptomic alterations of this model.

## Results

### Coexpression and coregulation of Magel2 and Necdin genes and generation of a mouse model with a deletion including both genes.

**Figure 1:**
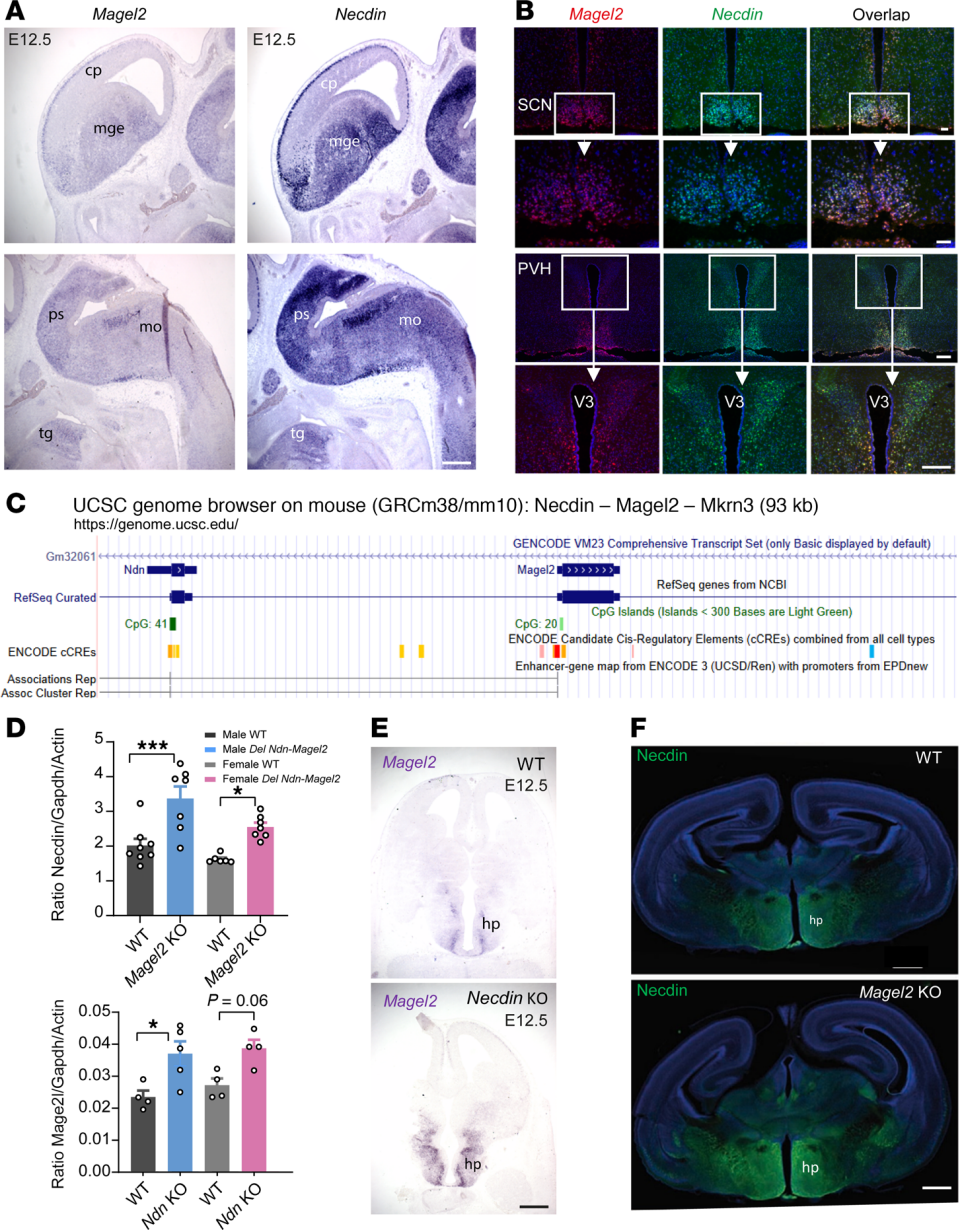
Coexpression and coregulation of *Necdin* and *Magel2* genes in the mouse brain. (**A** and **B**) Images showing *Necdin* and *Magel2* mRNA-expressing cells in the forebrain and brainstem of E12.5 mice (**A**) and in the hypothalamus of adult mice (**B**). (**C**) Map of the mouse genomic region including *Necdin* and *Magel2* genes, ENCODE *cis* regulatory elements, and associations between enhancers and promoters of genes, extracted from UCSC Genome Browser. A coregulation of *Magel2* and *Necdin* via shared enhancer is proposed (physical link). (**D**) qPCR analysis showing relative levels of *Necdin* and *Magel2* mRNAs in the hypothalamus of WT, *Magel2-*KO, or *Necdin-*KO male and female mice at P0 (*n* = 3–5 animals per group). (**E**) Images showing *Magel2* mRNA expression on horizontal brain sections (at the level of the presumptive hypothalamus) of WT and *Necdin-*KO embryos at E12.5. (**F**) Images showing Necdin immunoreactivity in coronal sections at the level of the hypothalamus of WT and *Magel2-*KO mice at P0. Data are presented as mean ± SEM. Statistical significance between groups was determined by a 2-way ANOVA with Šidák’s multiple-comparison test (**D**). *P < 0.05. Scale bars: 50 μm (**A** and **E**), 20 μm (**B**), 500 μm (**F**). cp, cortical plate; hp, hypothalamus; mge, median ganglion eminence; mo, medulla oblongata; ps, pons; tg, tongue; PVH, paraventricular nucleus; SCN, suprachiasmatic nucleus; V3, third ventricle.

We previously found that *Necdin* and *Magel2* mRNAs were highly expressed in the developing brain (12). Here, we showed a striking overlapping expression pattern of both genes in the embryonic and adult brain (Figure 1, A and B). We used a single-cell RNA-Seq–based (scRNA-Seq–based) interactive atlas (mousebrain.org) to examine which cell types expressed *Necdin* and *Magel2* and found that these genes were mainly expressed in neurons in various brain regions and neuronal systems and that nearly all *Magel2* cells also express *Necdin* (Supplemental Figure 1, A and B; supplemental material available online with this article; https://doi.org/10.1172/jci.insight.185159DS1). Our previous studies (12) and genomic analysis (https://genome.ucsc.edu) predicted that both genes share a common enhancer (Figure 1C). We have previously generated 2 mouse models in which the promoter and 5′ of the coding region of *Necdin* (*Ndn^tm1-Mus^*) (32) or *Magel2* (*Magel2^tm1-Mus^*) (20) were deleted, preventing the expression of *Necdin* and *Magel2* transcripts, respectively. In the present study, we used quantitative PCR (qPCR) and found that *Necdin* mRNA was overexpressed in the hypothalamus of *Magel2*-KO mice, and *Magel2* mRNA was overexpressed in *Necdin-*KO hypothalami at P0 (Figure 1D). We also showed by in situ hybridization that *Magel2* transcript was overexpressed in *Necdin-*KO brain (Figure 1E) and Necdin immunoreactivity was increased in the brains of *Magel2*-KO mice (Figure 1F). Together, these data show that *Magel2* and *Necdin* are spatiotemporally coregulated and share a putative enhancer with functions that partially overlap.

**Figure 2:**
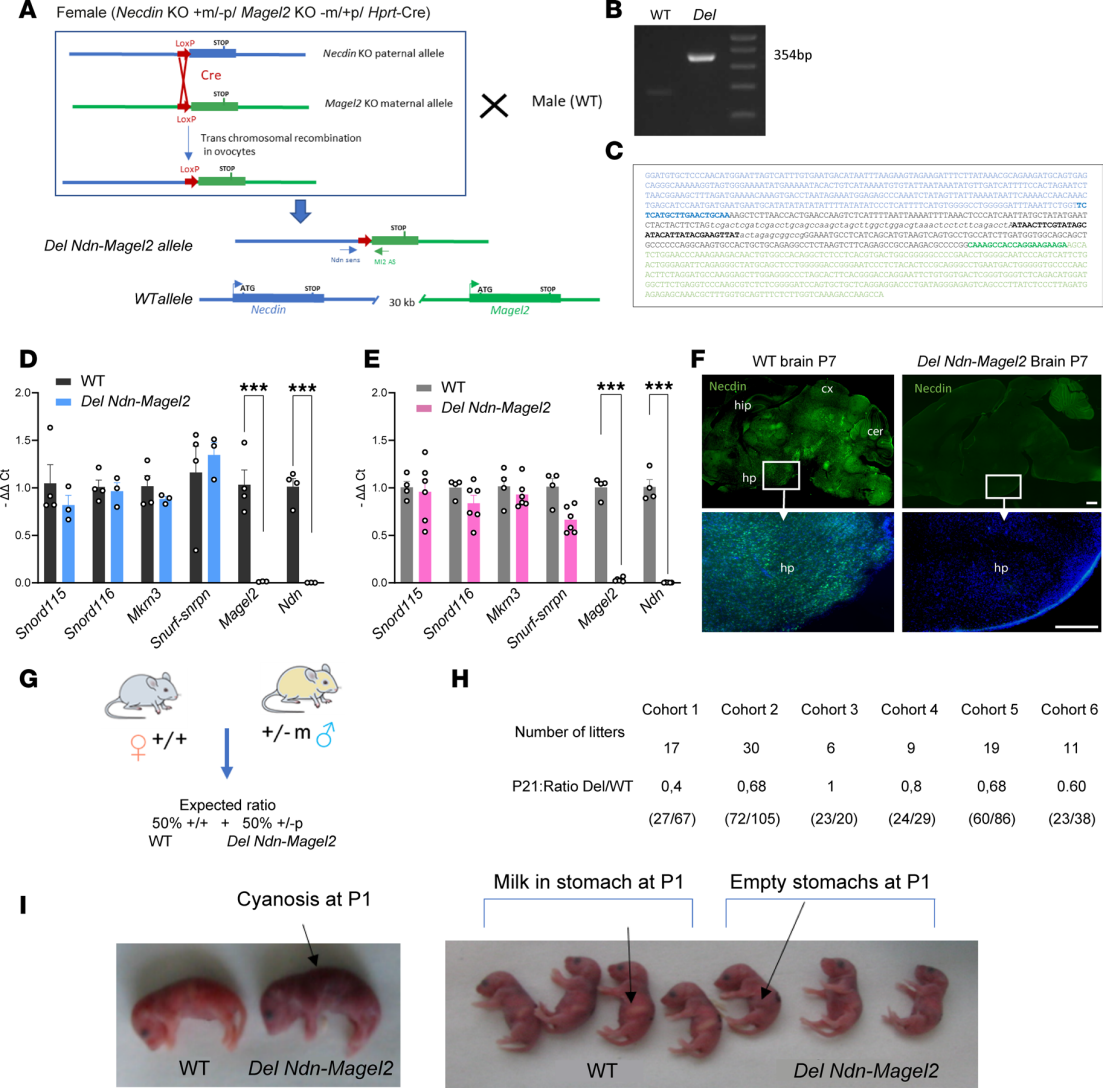
Construction and validation of the *Del Ndn-Magel2* mouse model. (**A**) Strategy to obtain a 32 kb deletion including both the *Necdin* and *Magel2* genes using a transallelic recombination approach. First, a female mouse containing 1 maternal allele with the *Magel2* deletion, 1 paternal allele with the *Necdin* deletion, and containing the *Hprt*-Cre gene was created using both *Necdin^tm1-Mus^*– and *Magel2^tm1-Mus^*–KO models and a transgenic mouse line expressing the Cre recombinase under Hprt promoter. We then crossed these female mice with WT male mice and screened the litter for the recombinant *Del Ndn-Magel2* allele using PCR with *Necdin* sense and *Magel2* antisense primers. The *Del Ndn-Magel2* allele being created in the ovocytes. (**B**) PCR product obtained from the recombinant *Del Ndn-Magel2* allele (354 bp) in the F1 generation. (**C**) Sequence of the recombined *Del Ndn-Magel2* allele with the *Necdin* upstream sequence (blue) and *Ndn* sense primer (bold blue), the LoxP sequence (bold black), and *Magel2* sequence (green), and Ml2 anti-sense primer (bold green). This sequence validates the recombination at the loxP sites. (**D** and **E**) qPCR analysis showing relative levels of *Snord115*, *Snord116*, *Mkrn3*, *Snurf-snron*, *Magel2*, and *Necdin* mRNAs in the hypothalamus of WT and *Del Ndn-Magel2* male (**D**) and female (**E**) mice at P60. (**F**) Images showing Necdin immunoreactivity on sagittal sections of WT and *Magel2-*KO mouse brains at P7. (**G**) Breeding strategy to generate *Del Ndn-Magel2* and WT litters with an expected ratio of 1 *Del Ndn-Magel2*/1 WT. (**H**) Ratio of *Del Ndn-Magel2* and WT mice at P21 from 6 cohorts produced in the different laboratories performing experiments for this study. (**I**) Photos showing cyanosis and lack of milk in the stomach associated to the death of *Del Ndn-Magel2* pups at P1. Data are presented as mean ± SEM. Statistical significance between groups was determined by a Mann-Whitney *U* test (**D** and **E**). ***P ≤ 0.002. Scale bar: 400 μm. cer, cerebellum; cx, cortex; hp, hypothalamus; hip, hippocampus.

Based on the findings described above, it appears that the combined loss of *Magel2* and *Necdin,* by reflecting more accurately the genetics of PWS, will be a more appropriate model to study a PWS-like phenotype. We, therefore, generated mice with a large deletion including both *Necdin* and *Magel2* genes (hereafter called *Del Ndn-Magel2* mice) using both *Ndn^tm1-Mus^* and *Magel2 ^tm1-Mus^*–single KO mice and an in vivo chromosomal rearrangement based on the Cre-loxP system (Figure 2A). We created a potentially novel allele with a 32 kb deletion that includes *Necdin*, the fragment between *Necdin* and *Magel2*, the *Magel2* promoter, and half of the *Magel2* coding part (Figure 2A). We screened and validated the recombined *Del Ndn-Magel2* allele by PCR using *Necdin* and *Magel2* primers, and we sequenced the recombined allele confirming a recombination at the loxP sites and the expected deletion (Figure 2, B and C). Because *Magel2* and *Necdin* are regulated by genomic imprinting resulting in transcriptional silencing of the maternal allele and expression of the paternal allele only, all studies described below were performed on heterozygous mice with a mutated paternal allele and a WT yet silent maternal allele (+m/–p), considered as KO. We measured *Magel2* and *Necdin* mRNA levels in the hypothalamus of *Del Ndn-Magel2* heterozygous male and female mice (+m/–p) using qPCR and confirmed the loss of expression of both transcripts (Figure 2, D and E). We also confirmed the loss of Necdin protein expression in *Del Ndn-Magel2* brains using IHC (Figure 2F). The loss of Magel2 protein expression could not be checked due the lack of specific MAGEL2 antibodies. Because all PWS genes share a common spatiotemporal and imprinted regulation, it was possible that the genomic deletion of *Necdin* and *Magel2* could also have an effect on the transcriptional regulation of other PWS genes. However, we did not find significant alterations in the expression of *Snord115, Snord 116*, *Mkrn3*, or *Snurf-snrpn* in the hypothalamus of *Del Ndn-Magel2* mice (Figure 2, D and E).

We generated several cohorts of *Del Ndn-Magel2* heterozygous and WT mice in different laboratories. Although the expected Mendelian ratio of our cross was 1:1, we often observed a reduced number of *Del Ndn-Magel2* mice compared with WT animals (Figure 2G). In 5 of 6 cohorts generated (representing 92 litters), we found a reduction of 40% (ranging from 20% to 60%) of *Del Ndn-Magel2* mice compared with WT animals (Figure 2H). The variability in the postnatal lethality observed in *Del Ndn-Magel2* pups appeared to be correlated with the level of sanitary status of the animal facility in which animals were housed; when animals were housed in a pathogen-free animal facility and fed sterile food, the phenotype was less severe (with 0% lethality) than when animals were housed in a conventional animal facility (with 60% lethality in mutant mice versus control littermates). The lethality occurred during the first day of life. Monitoring neonates shortly after birth revealed cyanosis in dead *Del Ndn-Magel2* mice (Figure 2I), suggesting a lack of proper oxygenation due to respiratory dysfunction as observed in *Ndn^tm1-Mus^*–KO mice (38, 43). Dead *Del Ndn-Magel2* neonates also lacked milk in their stomachs (Figure 2I) as previously observed in the *Magel2^tm1-Mus^*–KO mouse model (20). Together, these data indicate that the possible causes of death in *Del Ndn-Magel2–*KO newborns include respiratory defects and/or feeding deficits at birth.

### Del Ndn-Magel2 mice develop sensory alterations in infancy and social exploration deficits during adulthood.

Because patients with PWS present symptoms at birth evolving with age, we began to characterize the phenotype of *Del Ndn-Magel2* during neonatal life. We first used 11 reliable tests established by Roubertoux et al. (44) to assess the sensory and motor abilities during the first 2 weeks of postnatal life (Supplemental Figure 2A). Each test was performed at specific postnatal ages defined by the critical period during which the response is established in 100% of control mice in the mouse strain that we used (i.e., C57BL6/J) (Supplemental Figure 2B). We compared *Del Ndn-Magel2* with WT pups from the same litters. Since we did not observe differences between males and females, we pooled both sexes.

**Figure 3:**
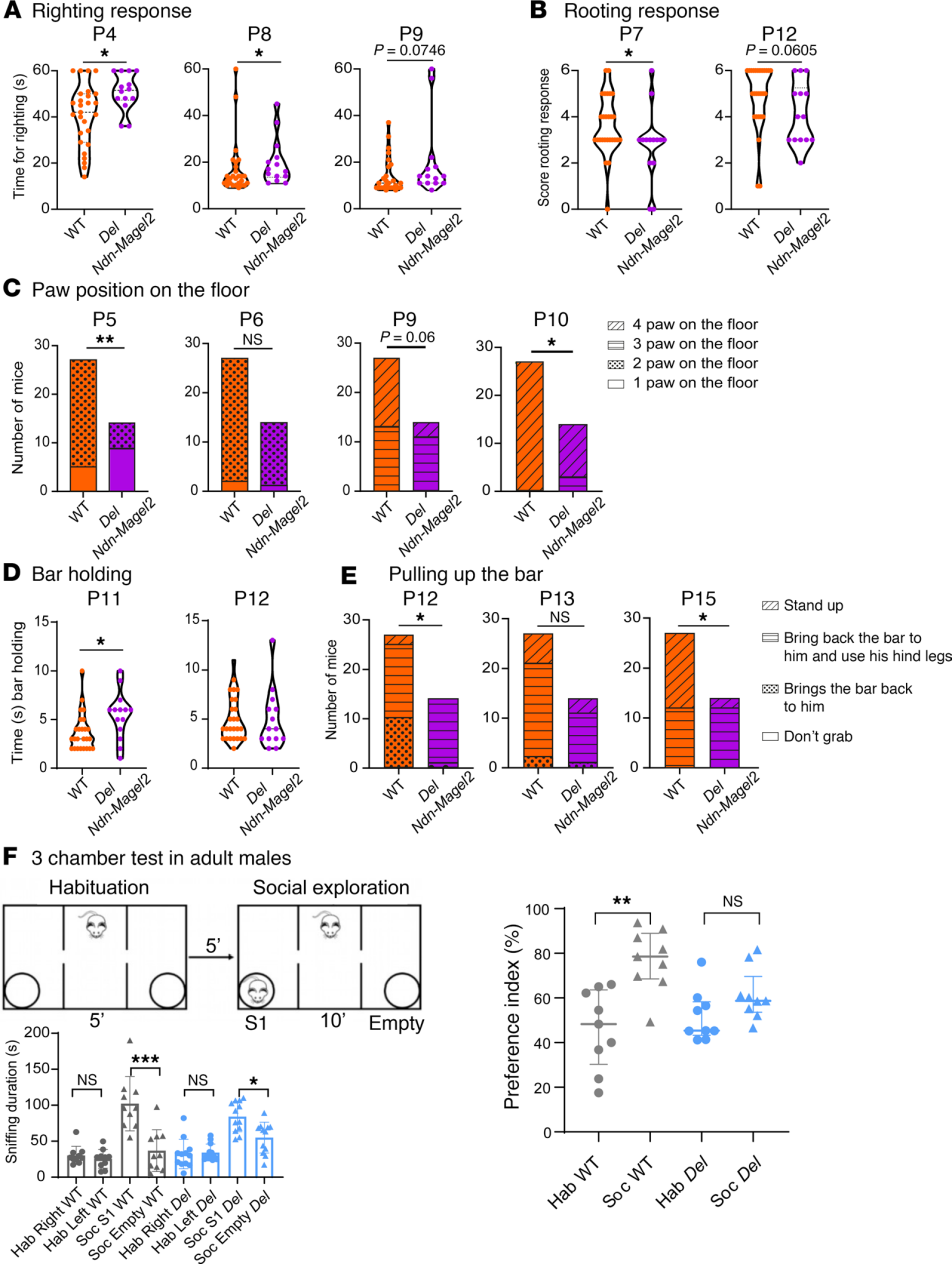
*Del Ndn-Magel2* mice display early sensory alterations and social exploration deficits during adulthood. (**A**) Righting response in WT and *Del Ndn-Magel2* mice at P4, P8, and P9. (**B**) Rooting response in P7 and P12 WT and *Del Ndn-Magel2* pups. (**C**) Paw position test in WT and *Del Ndn-Magel2* mice at P5, P6, P9, and P10. (**D**) Holding bar test in P11 and P12 WT and *Del Ndn-Magel2* pups. (**E**) Test assessing the pulling up on the bar after hanging in P12, P13, and P15 WT and *Del Ndn-Magel2* pups (*n* = 14–27 animals per group). These tests evaluate sensory motor abilities. (**F**) Three-chamber test in adult WT and *Del Ndn-Magel2* mice (*n* = 10–12 animals per group) reporting the interaction time (i.e., sniffing duration in seconds) between mice measured during habituation (Hab, left or right empty grid) or in the context of social exploration (Soc, empty grid versus novel mouse S1). Histogram on the right report the preference index (sniffing duration/sniffing duration of the novel mouse + sniffing duration of the empty grid). Data are presented as mean ± SEM. Statistical significance between groups was determined by a Mann-Whitney *U* test (**A**, **B**, and **D**), a χ^2^ test (**C** and **E**), or a repeated measures 1-way ANOVA with Šidák’s multiple-comparison test (**F**). *P < 0.05, **P < 0.01, ***P < 0.001.

We found differences between mutant and WT mice in 5 of the 11 tests. *Del Ndn-Magel2* pups were less efficient in righting response, rooting response, and paw position on the floor (Figure 3, A–C). The righting response reveals motor and sensory (proprioception) abilities, and its development was delayed in *Del Ndn-Magel2* pups at P4 and P8 (Figure 3A). The rooting reflex involves cranial sensory nerves; this reflex disappeared in WT mice across postnatal development, but it was more frequent in *Del Ndn-Magel2* pups at P7 and at P12 (tendency) compared with WT mice (Figure 3B). The paw position test is mainly a sensory test (proprioception, touch) and revealed a delay in sensory development in *Del Ndn-Magel2* pups (Figure 3C). In contrast, mutant mice performed better in the bar-holding test at P11 (Figure 3D). This is a motor test, requiring muscle strength. Interestingly, *Del Ndn-Magel2* pups also performed better in the pulling up on bar test at P12 (Figure 3E) — i.e., they were more efficient in bringing back the bar to them and using their hind legs. However, they were less efficient in standing up on the bar at P15, requiring more sensory abilities (Figure 3E). However, mutant and WT pups performed similarly in the vertical climbing test as well as the 2 other tests involving vestibular and motor activity (i.e., climbing the slope and cliff avoidance) (Supplemental Figure 3, C–E). Similarly, the age of eye opening and auditory canal opening, which normally occurs between P13 and P14, was similar between *Del Ndn-Magel2* and WT mice (Supplemental Figure 3 A and B). Altogether, these results indicate that *Del Ndn-Magel2* pups presented a delay in sensory (tactile and proprioception) maturation but not in motor abilities, as they actually displayed an increased muscular strength.

We performed a series of behavioral tests in adult mice that were previously done in the *Magel2-*KO mouse model. No differences were observed between *Del Ndn-Magel2* and WT mice in the motor activity, anxiety or spontaneous activity (Supplemental Figure 4 and Supplemental Methods). In the object-recognition test, if the animals were close to the novel object, *Del Ndn-Magel2* mice showed a greater interest in the novelty (sniffing time) than the familiar object (Supplemental Figure 4E). However, when animals were far from to the novel object, mutant mice did not preferentially move toward the novel object (Supplemental Figure 4E), suggesting altered recognition or interest. Sociability was evaluated in male mice only, by testing the preference for a congener placed in a wire cage as compared with an empty wire cage (3-chamber test, social recognition task). WT and *Del Ndn-Magel2* mice spent more time interacting with a congener compared with the empty grid (Figure 3F, sniffing duration). However, the index of sociability was significantly lower in *Del Ndn-Magel2* mice and is even similar to the index of habituation (Figure 3F) suggesting an alteration of social recognition (and sociability) in *Del Ndn-Magel2* compared with WT mice.

### Effect of the combined deletion of Magel2 and Necdin on body weight, body composition, energy, and glucose homeostasis.

**Figure 4:**
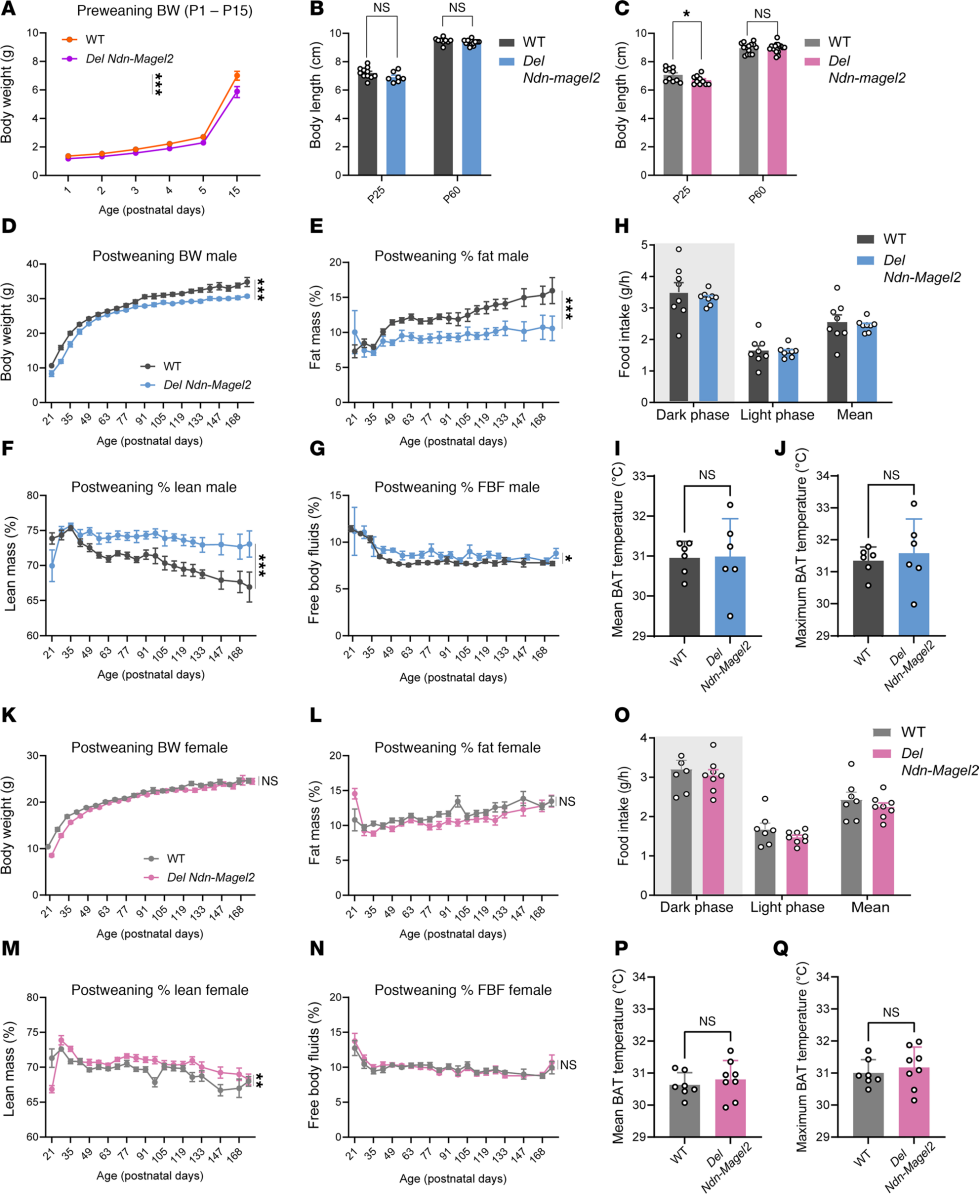
Sexually dimorphic effects of *Magel2* and *Necdin* deficiencies on growth curves and body composition. (**A**) Body weight from birth (P0) to P15 of *Del Ndn-Magel2* and WT mice (*n* = 13–28 animals per group). (**B** and **C**) Body length at P24 and adulthood (P60) of male (**B**) and female (**C**) *Del Ndn-Magel2* and WT mice (*n* = 7–13 animals per group). (**D**–**N**) Body weight (**D** and **K**), fat mass (**E** and **L**), lean mass (**F** and **M**), and free body fluids (**G** and **N**) of male (**D**–**G**) and female (**K**–**N**) *Del Ndn-Magel2* and WT mice from weaning (P21) to P168 (*n* = 13–18 animals per group). (**H**–**Q**) Food intake (**H** and **O**) and mean (**I** and **P**) and maximum (**J** and **Q**) brown adipose tissue temperature in male (**I** and **J**) and female (**P** and **Q**) *Del Ndn-Magel2* and WT mice at P120 (*n* = 6 animals per group). Data are presented as mean ± SEM. Statistical significance between groups was determined by Mixed-effect test (**A**, **D**–**G**, and **K**–**N**), Student’s *t* test (**B**, **C**, **H**, **K**, **O**, and **Q**) or a Mann-Whitney *U* test (**I** and **J**). *P ≤ 0.033, **P ≤ 0.002, ***P ≤ 0.0002.

To evaluate the metabolic consequences of the loss of *Magel2* and *Necdin*, we first measured the body weight of *Del Ndn-Magel2* and WT mice throughout postnatal life. Mutant mice displayed a lower body weight, starting at P1 and continuing until P15 (Figure 4A). Notably, the difference in body weight at weaning in female *Del Ndn-Magel2* mice was associated with a smaller body length (Figure 4B) and was specific to the preweaning period, as *Del Ndn-Magel2* females had normal body weight curves and body length after weaning, although they were leaner (Figures 4, C, K, and M). In contrast, male *Del Ndn-Magel2* mice displayed lower body weights but a normal body size from weaning (P21) to 6 months of age (P168) (Figure 4, B and D), with changes in body composition, as revealed by a lower fat mass and a higher lean mass compared with WT mice (Figure 4, E–G). However, we did not find differences in fat mass in female mutant mice (Figure 4L). Food intake (even when normalized to body weight) was comparable between WT and mutant male and female mice (Figure 4, H and O). Also, total body fluids (Figure 4, G and N) and the mean and maximal brown adipose tissue temperature was normal in male and female *Del Ndn-Magel2* mice (Figure 4, I, J, P, and Q).

**Figure 5:**
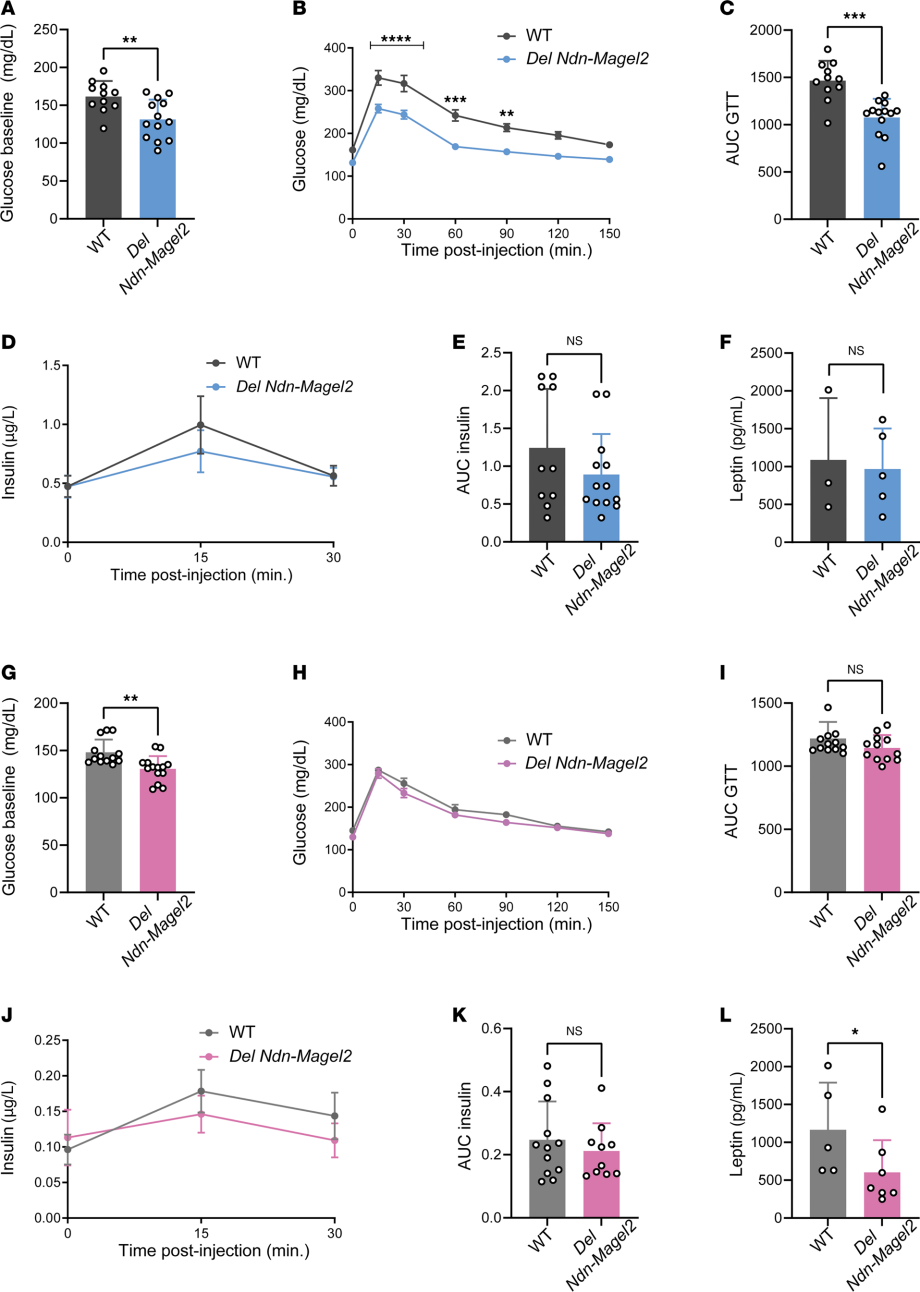
The combined loss of *Magel2* and *Necdin* alters glucose metabolism in males. (**A**–**K**) Basal glycemia (**A** and **G**), glucose tolerance tests (GTT) (**B** and **H**), areas under the GTT curve (**C** and **I**), serum insulin levels during GTT (**D** and **J**), and AUC (**E** and **K**) in male (**A**–**E**) and female (**G**–**K**) *Del Ndn-Magel2* and WT mice at P130 (*n* = 11–13 animals per group). (**F** and **L**) Serum Leptin concentration in male (**F**) and female (**L**) *Del Ndn-Magel2* and WT mice at P160–P240 (*n* = 3–4 animals per group). Data are presented as mean ± SEM. Statistical significance between groups was determined by Mann-Whitney *U* test (**A**, **E**, **G**, **I**, **K**, and **L**), or 2-way ANOVA with the Geisser-Grennhouse correction following by a Tukey’s multiple-comparison test (**B** and **H**) or a Sidak’s multiple-comparison test (**D** and **J**). *P ≤ 0.033, **P ≤ 0.002, ***P ≤ 0.0002, ****P ≤ 0.0001

It is known that body composition is a factor that could influence glucose regulation, and it has been reported that patients with PWS have improved glucose metabolism (45). We, therefore, measured several indices of glucose homeostasis in *Del Ndn-Magel2* and WT mice. Both male and female *Del Ndn-Magel2* mice exhibited lower fasting glycemia levels than their respective littermates (Figure 5, A and G). Additionally, male, but not female, *Del Ndn-Magel2* mice displayed an improved glucose tolerance after a glucose challenge (Figure 5, B, C, H, and I). The increased glucose tolerance appeared independent of improved insulin secretion, as *Del Ndn-Magel2* mice had normal insulin levels during the glucose-tolerance test (Figure 5, D, E, J, and K). Mutant mice also displayed normal leptin levels (Figure 5, F and L).

We also conducted a comprehensive assessment of the energy balance regulation. No alterations energy expenditure, respiratory exchange ratio (RER), or xy locomotor activity were found in either male or female *Del Ndn-Magel2* mice compared with their WT littermates (Supplemental Figure 5, A–F). Similarly, metabolic responses to fasting and refeeding appeared normal in mutant mice (Supplemental Figure 6). Nevertheless, the combined loss of *Magel2* and *Necdin* impacted z-rearing behavior, with a greater frequency of z-rearing during the light phase in male, but not female, *Del Ndn-Magel2* mice (Supplemental Figure 5, G and H).

### Delayed puberty onset in Del Ndn-Magel2 mice.

**Figure 6:**
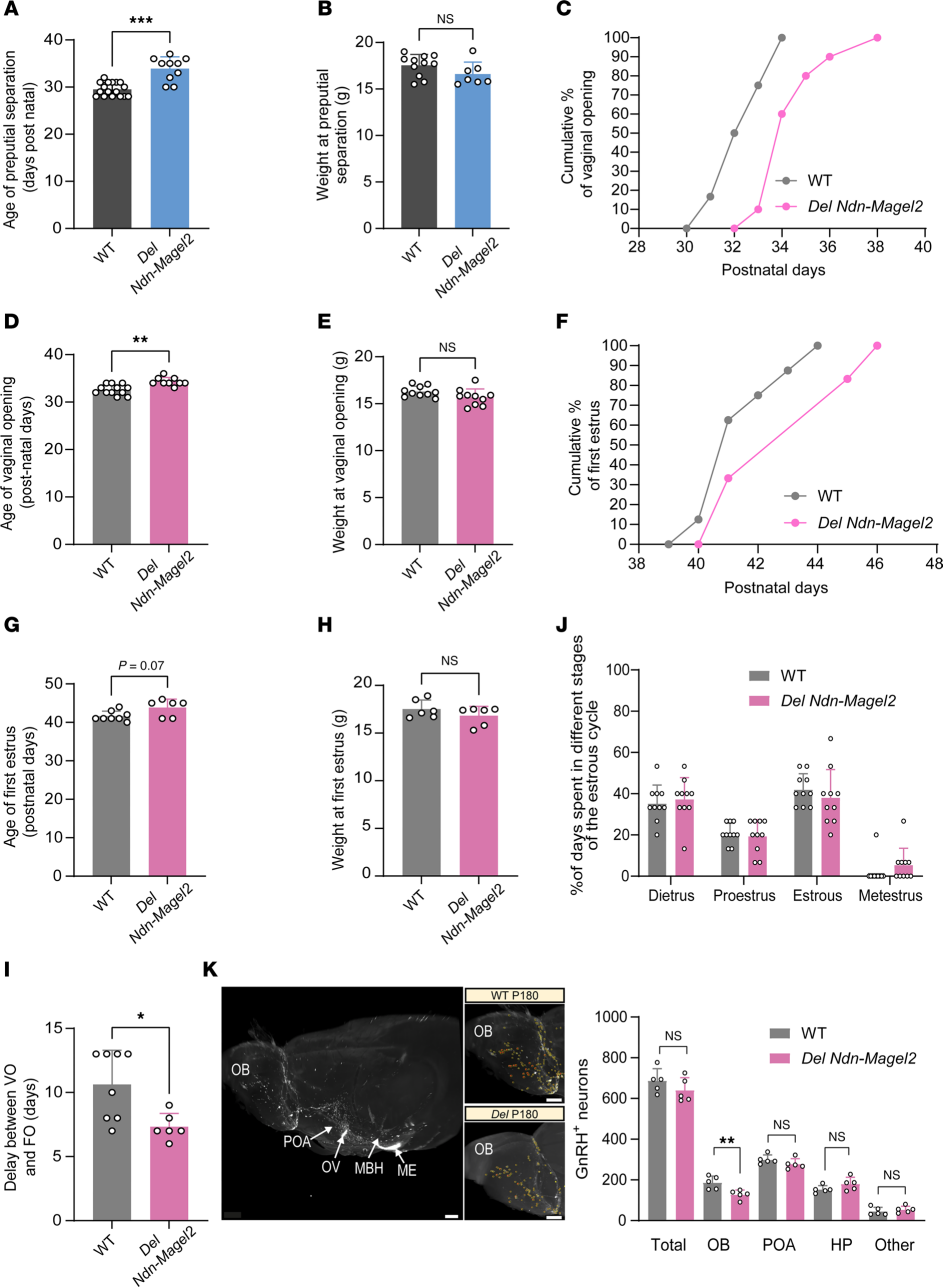
Delayed the onset of puberty in *Del Ndn-Magel2* mice. (**A** and **B**) Age and weight of balano-preputial separation in male *Del Ndn-Magel2* KO or WT mice (*n* = 18–20 animals per group). (**C**–**J**) Cumulative percentage data for vaginal opening (VO) (**C**), age (**D**) and weight (**E**) of vaginal opening, cumulative percentage data of first estrus (**F**), age (**G**) and weight (**H**) of first estrus, delay between VO and first estrus (**I**), and percentage of days spent in different stages of the estrous cycle (**J**) in female *Del Ndn-Magel2* KO or WT mice (*n* = 10–12 animals per group). (**K**) Representative image of cleared brains and immunolabeling for GnRH quantification of the number of GnRH-immunoreactive neurons in female *Del Ndn-Magel2* or WT mice at P180 (*n* = 5 animals per group). Data are presented as mean ± SEM. Statistical significance between groups was determined using a Mann-Whitney *U* test (**A**–**J**). **P ≤ 0.033, **P ≤ 0.002, ***P ≤ 0.0002. Scale bar: 500 μm. MBH, mediobasal hypothalamus; ME, median eminence; OB, olfactory bulb; OV, organum vasculosum of the lamila terminalis; POA, preoptic area.

Hypogonadism and delayed puberty are often reported in patients with PWS (1). We then characterized sexual maturation in *Del Ndn-Magel2* mice by monitoring the age of balano-preputial separation in males and the age of vaginal opening and first estrus in females. Balano-preputial separation occurred at 29.5 ± 0.3 days in WT males, but it was observed at 33.9 ± 0.8 days in *Del Ndn-Magel2* males (Figure 6A). Notably, the weight at balano-preputial separation was comparable between WT and mutant mice (Figure 6B). In females, the age of vaginal opening in WT animals was observed at 32.6 ± 0.3 days, but it was delayed at 34.3 ± 0.3 days in *Del Ndn-Magel2* mice (Figure 6, C and D). However, the weight at vaginal opening, age, and weight of first estrus was normal in *Del Ndn-Magel2* mice (Figure 6, E–H). These results show that sexual maturation is delayed in *Del Ndn-Magel2* KO mice of both sexes. Accordingly, there was a significant reduction in the delay between the vaginal opening and the first ovulation, indicative of altered timing of puberty onset in female *Del Ndn-Magel2* mice (Figure 6I). During adult life, *Del Ndn-Magel2* mice displayed regular estrous cycles (Figure 6J). Because GnRH neurons are critical regulators of reproductive function (46), we also investigated the distribution of GnRH neurons in the brain of *Del Ndn-Magel2* mice and their WT littermates using the iDISCO 3D approach (47). The overall distribution and total number of GnRH neurons were comparable between WT and mutant mice (Figure 6K). However, we found fewer GnRH neurons in the olfactory bulb of *Del Ndn-Magel2–*KO mice (Figure 6K).

### Neuroanatomical alterations in the hypothalamus of Del Ndn-Magel2 mice.

**Figure 7:**
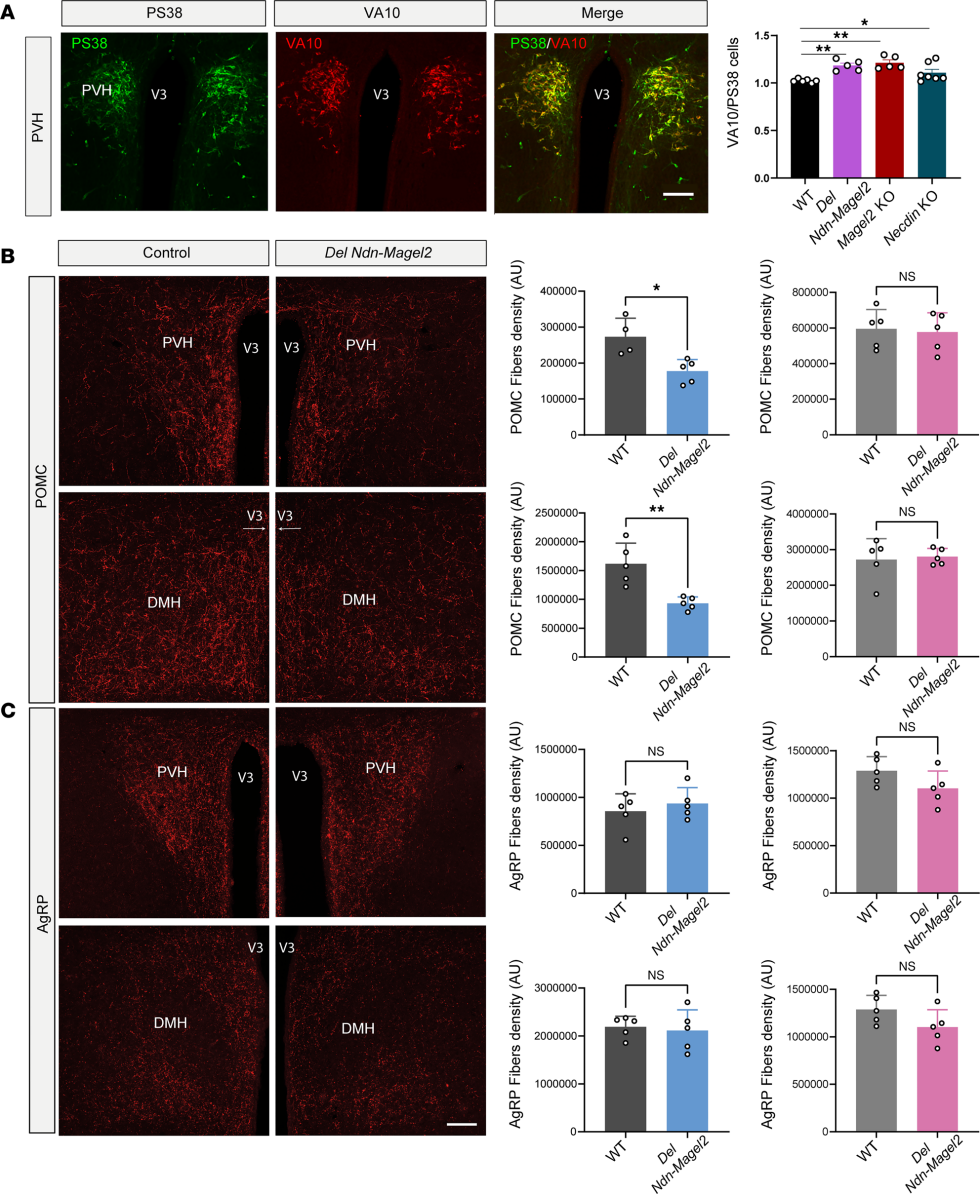
Disruption of hypothalamic melanocortin circuits in *Del Ndn-Magel2* mice. (**A**) Confocal images and quantification of the ratio of PS38- and VA10-immunoreactive neurons in the paraventricular nucleus (PVH) of P0 *Del Ndn-Magel2* and WT mice. (**B** and **C**) Confocal images and quantification of the density of POMC- (**B**) and AgRP-immunoreactive (**C**) fibers innervating the paraventricular and the dorsomedial (DMH) nuclei of the hypothalamus of male (blue) and female (pink) *Del Ndn-Magel2* KO or WT mice at P98 (*n* = 5 animals per group). Data are presented as mean ± SEM. Statistical significance between groups was determined by a mixed-effect test (**A**) or a Mann-Whitney *U* test (**B** and **C**). *P ≤ 0.033, **P ≤ 0.002. Scale bar: 100 μm. V3, third ventricle.

Previous histopathological studies in the hypothalamus of adult patients with PWS reported a significant reduction in the number of OT neurons (48, 49). However, these studies utilized antibodies against the mature form of OT, bringing into question whether the reduced number of OT-immunopositive neurons is truly due to a loss of OT neurons or is caused by perturbations in peptide maturation. Therefore, we used 2 antibodies targeted against the prohormone (i.e., the PS38 antibody) or the intermediate forms of the neurohormone (i.e., the VA10 antibody). We then analyzed the ratio of neurons expressing the intermediate form of OT with those expressing the prohormone and observed an accumulation of the intermediate form of the neuropeptide in *Magel2*-KO, *Necdin*-KO, and *Del Ndn-Magel2* mice at P0 (Figure 7A).

The melanocortin system is a critical component of hypothalamic pathways regulating metabolism. We previously reported a disruption of melanocortin circuits in *Magel2*-KO mice (the *Magel2*^tm1Stw^ line) with a reduced density of POMC-immunoreactive fibers in the paraventricular nucleus of *Magel2*-KO male mice compared with WT mice but a normal density of agouti-related peptide–orexigenic (AgRP-orexigenic) fibers (29). Consistent with these findings, we found a 1.5-fold and a 1.7-fold reduction in the density of POMC-immunoreactive fibers in the PVH and DMH, respectively, in male but not female *Del Ndn-Magel2* mice compared with WT littermates (Figure 7B). The density of AgRP-immunoreactive fibers was normal in the PVH and DMH of male and female mutants (Figure 7C).

### Respiratory alterations in Del Ndn-Magel2 mice.

**Figure 8:**
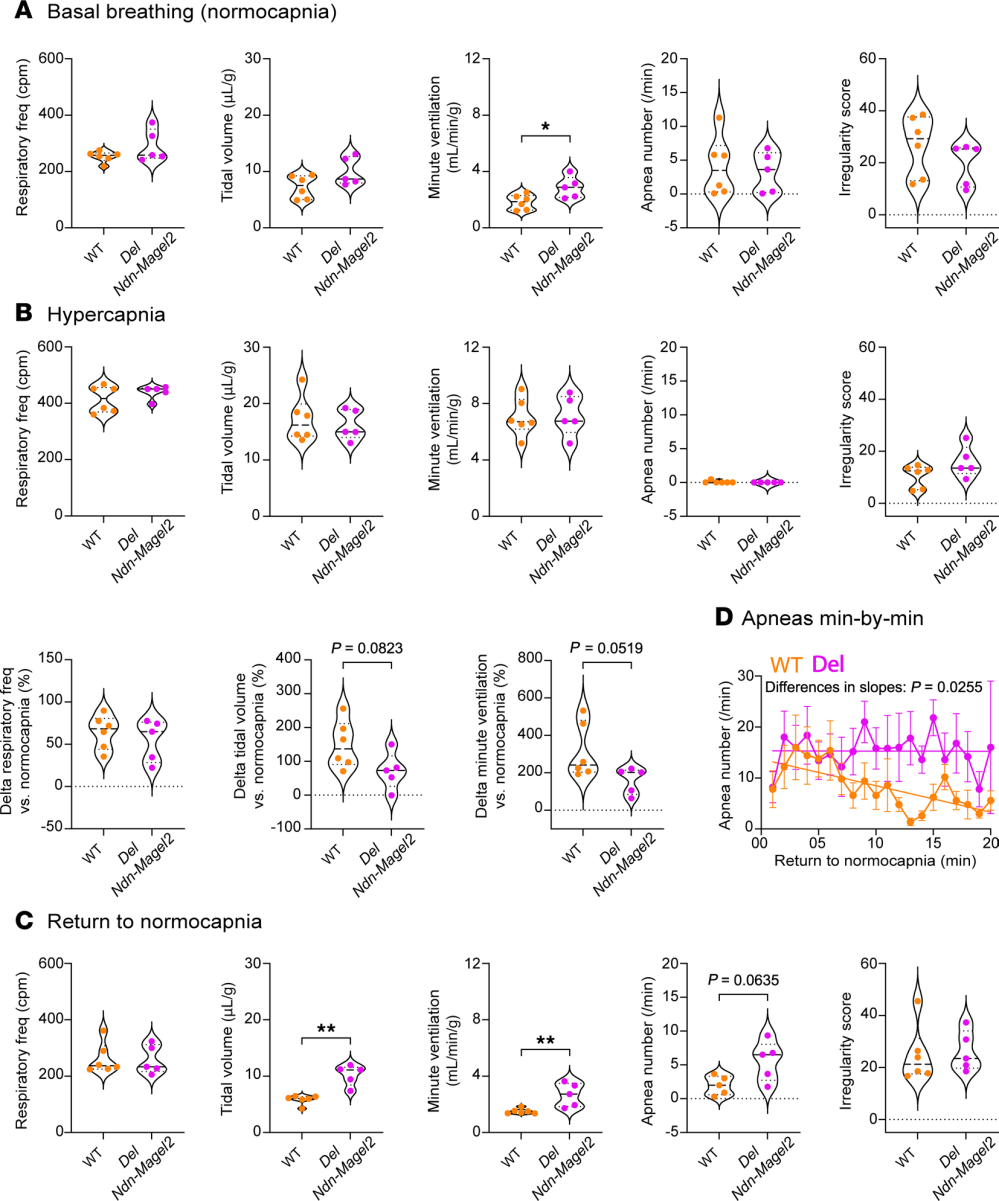
Increased ventilation and posthypercapnic apneas in *Del Ndn-Magel2* mice. (**A**–**C**) Analysis of the breathing activity in P30 *Del Ndn-Magel2* (*n* = 5 animals per group) and WT (*n* = 6 animals per group) mice using in vivo whole-body plethysmography during basal conditions (normocapnia) (**A**), during a 10 minute hypercapnic challenge (4% CO_2_) (**B**), and during the first 20 minutes of return to normocapnia (**C**) following the hypercapnic challenge. (**D**) Analysis of the number of apneas per minute during the first 20 minutes of return to normocapnia. Statistical significance between groups was determined by a Mann-Whitney *U* test (**A**–**C**) or a linear fitting with the least squares regression method, extra sum-of-squares F test to compare slope differences (**D**). *P < 0.05, **P < 0.001. cpm, cycles per minute.

Respiratory distress was reported in *Necdin*-KO neonates and adults (33, 38). The breathing activity of *Del Ndn-Magel2* mice and WT littermates was assessed at P30 using in vivo whole-body plethysmography (33). During quiet breathing in normocapnia, *Del Ndn-Magel2* mice displayed an increased minute ventilation compared with WT mice, mainly due to a tendency for an increased tidal volume, while respiratory frequency was similar in both groups (Figure 8A). There was no difference in the number of apneas and in the irregularity of breathing between WT and *Del Ndn-Magel2* mice. A 10 minute hypercapnic challenge (4% CO_2_) increased ventilatory parameters to similar levels in both *Del Ndn-Magel2* and WT mice (Figure 8B). There was a tendency for a smaller Δ increase in minute ventilation and tidal volume in *Del Ndn-Magel2* mice compared with WT mice. During the first 20 minutes of return to normocapnia following the hypercapnic challenge, the *Del Ndn-Magel2* mice had increased minute ventilation and increased tidal volume compared with WT mice, similar to during basal breathing before the hypercapnic challenge (Figure 8C). Also, during this period, *Del Ndn-Magel2* mice showed an overall tendency for more apneas (Figure 8C). This was due to a persistence of the posthypercapnic increase in apneas throughout the first 20 minutes of return to normocapnia in mutant mice, as WT mice showed a reduction in their number of apneas starting approximately 5 minutes after the return to normocapnia (Figure 8D). These results indicate an increased ventilation of *Del Ndn-Magel2* mice in basal normocapnic condition and a persistent increase in apneas following a hypercapnic challenge.

### Hypothalamic genes are differentially expressed in Del Ndn-Magel2 mice during the circadian cycle.

**Figure 9:**
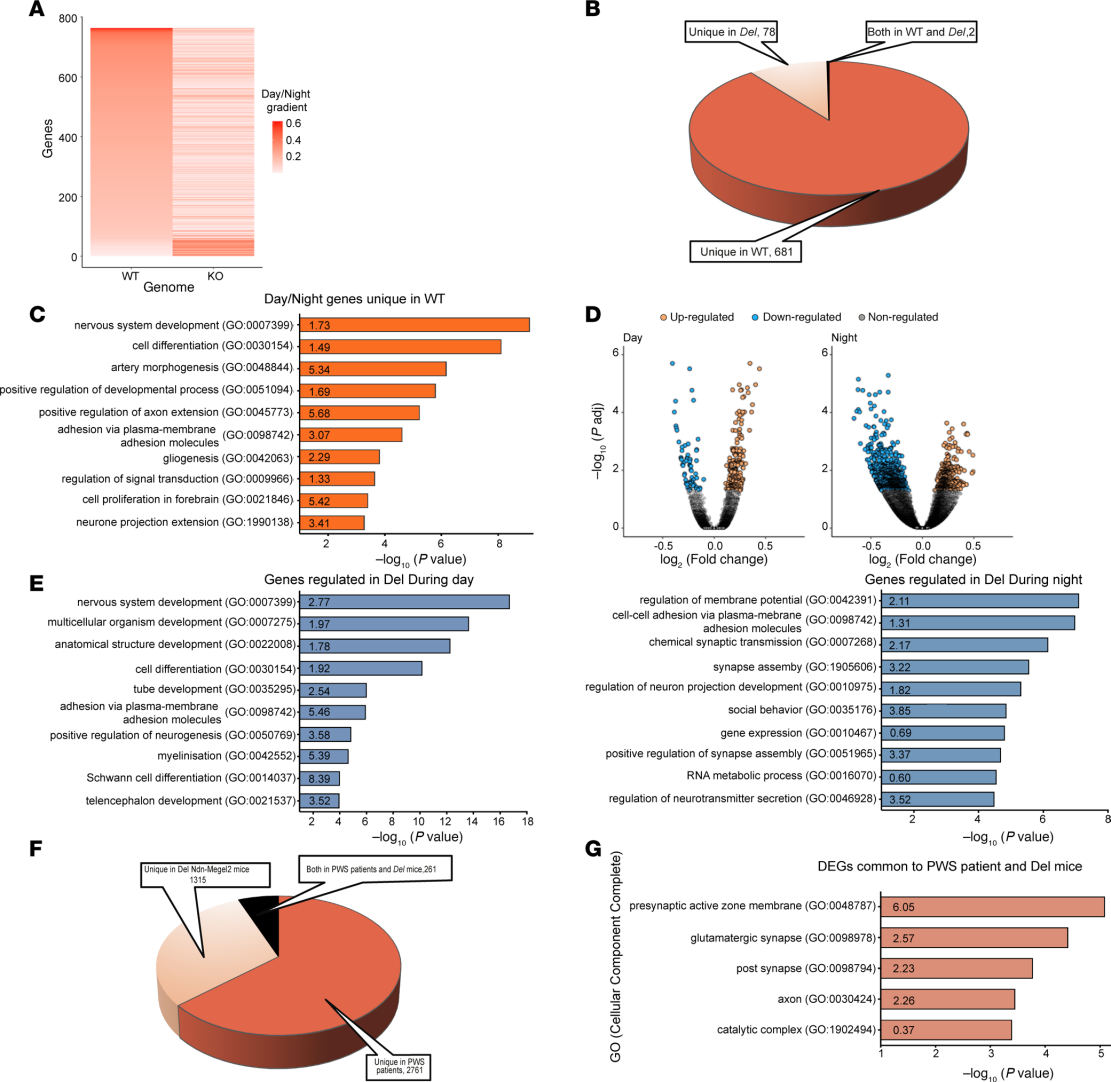
Transcriptomic analysis of genes that are dysregulated in the hypothalamus of *Del Ndn-Magel2* mice. (**A**) Heatmaps representing the gradient of change (as the absolute value of log_2_ fold change) between day and night for all expressed genes in WT (left part) and for corresponding genes displayed in the same order in *Del Ndn-Magel2* mice (right part) at P70–P98 (*n* = 5 animals per group). (**B**) Effect of *Magel2*-*Necdin* double deletion on differentially day/night expressed genes. In total, 681 genes differentially expressed between day and night in WT lose their differential day/night expression in KO (Unique in WT, 90%), while 78 genes acquire a de novo day/night difference in expression (Unique in KO, 10%). Only 2 genes display a differential day/night expression in both mouse lineages (both in WT and KO). (**C**) Functional characterization by Panther analysis of genes belonging to the category “Unique in WT” showing the top 10 GO biological processes with the highest *P* value. The numbers inside the columns correspond to the fold enrichment. (**D**) Volcano plots illustrating upregulated (orange points) and downregulated (blue points) genes in *Del Ndn-Magel2* versus WT mice during the day (left part) and during the night (right part). During the day, 74% of genes are upregulated in *Del Ndn-Magel2*mice while 68% are downregulated during night. (**E**) Functional characterization by Panther analysis of genes affected in *Del Ndn-Magel2* mice during the day (left part) and during the night (right part) showing the top 10 GO biological processes with the highest *P* value. The numbers inside the columns correspond to the fold enrichment. (**F**) Diagram showing genes that are commonly dysregulated in the hypothalamus of *Del Ndn-Magel2* mice and patients with PWS (48). (**G**) Functional characterization by Panther analysis of genes commonly affected in the hypothalamus of *Del Ndn-Magel2* mice and patients with PWS showing the top 5 GO biological processes with the highest *P* value.

Transcriptional studies have proven helpful in understanding disease mechanisms. Therefore, we performed bulk RNA-Seq on the hypothalamus of adult *Del Ndn-Magel2* mice and their WT littermates. Since *Magel2* and *Necdin* have a circadian expression, with an approximate 1.5-fold increase in expression level during the night versus day (Figure 9A), and it has been shown dysregulation of the circadian activity and circadian genes (36, 50) in single *Necdin-* and *Magel2*-KO, we investigated genes that were differentially expressed between the middle of day (ZT6) and the middle of the night (ZT18) in the hypothalamus of *Del Ndn-Magel2* versus WT littermates. In the hypothalamus of WT mice, we found 683 genes that were differentially expressed between the ZT6 and ZT18 (Figure 9B). It may be assumed that these genes display a rhythmic pattern over the nychthemeral cycle. Remarkably, 681 of these 683 genes lose their differential expression between day and night in the *Del Ndn-Magel2* mice (Figure 9, B and C). Because measurements were taken at only 2 time points within the nychthemeral cycle, it was not possible to determine whether these genes became arrhythmic or if their phase had been shifted. Nevertheless, a Panther analysis revealed that, in this list of 681 genes that displayed a differential day/night expression only in WT mice, the terms ‘‘nervous system development,” “cell differentiation,” and “cell-cell adhesion” were among the most significant enriched annotations in Gene Ontology Biological Processes (GO-BP) (Figure 9C). We also identified 78 genes in the hypothalamus of *Del Ndn-Magel2* mice that acquired a day-night difference that was not found in WT mice (Figure 9, A and B). As previously highlighted, it cannot be determined whether these genes became rhythmic when they were not in the WT mice or if a phase shift enables the detection of a day/night difference that was undetectable with 2 measurement times in the WT. Overall, it appeared that 99% of genes with a differential day/night expression in both WT and *Del Ndn-Magel2* mice were dependent on *Magel2/Necdin* expression. These genes could be classified into 3 different categories depending on whether (a) they lost their differential expression, (b) they had a modified rhythmic pattern, or (c) they acquired a differential day/night expression (Figure 9, B and C).

We further analyzed the effect of the combined deletion of *Magel2* and *Necdin* on hypothalamic gene expression during the day (ZT6) and the night (ZT18). During the day, 261 genes had a different expression level in *Del Ndn-Magel2* compared with WT mice, with 68 (26%) downregulated and 193 (74%) upregulated (Figure 9D). As shown after Panther analysis, these 261 genes affected by *Magel2*/*Necdin* deletion were shown to be significantly associated with different functions such as “development,” “differentiation,” and “myelination” (Figure 9E). At night, more genes (1,295 genes) had their expression level differentially regulated in *Del Ndn-Magel2* mice, but while 874 (68%) of these genes displayed a downregulation, 421 genes (32%) were upregulated (Figure 9E). These 1,295 regulated genes were mostly associated with synaptic transmission, cell-cell adhesion, and social behavior (Figure 9E). Among all regulated genes either during the day or during the night (1,556 genes), 258 were found to display a day/night differential expression in WT, meaning that around 17% of genes whose expression was altered in *Del Ndn-Magel2* mice could be circadian rhythmic genes (data not shown).

We also explored whether there were genes that were commonly dysregulated in *Del Ndn-Magel2* mice and patients with PWS by comparing our transcriptomic data with an RNA-Seq analysis previously performed in the hypothalamus of patients with PWS (48). We found that 261 genes were commonly dysregulated in both *Del Ndn-Magel2* mice and patients with PWS (Figure 9F and Supplemental Table 1). In total, 1,315 genes were dysregulated only in *Del Ndn-Magel2* mice, and 2,761 were dysregulated only in patients with PWS (Figure 9F). A Panther analysis revealed that, in this list of 261 genes that were commonly dysregulated in mice and humans, the terms ‘‘presynaptic active zone membrane,” “glutamatergic synapse,” “post synapse,” and “axon” were among the most significant enriched annotations in GO-BP (Figure 9G).

## Discussion

The present study describes the phenotypic characterization of a mouse model with a deletion of both *Magel2* and *Necdin,* 2 genes also being deleted in PWS. The rationale for generating this mouse model was to avoid a potential functional redundancy between these 2 genes that could mask some symptoms in the *Necdin* or *Magel2*–single KO mice, both of which are genes encoding MAGE proteins that bind E-3 ubiquitin ligases specifying proteins for ubiquitination (51). Firstly, we confirmed that *Magel2* and *Necdin* are coexpressed in many brain nuclei throughout life and are coregulated, potentially sharing a common enhancer. More importantly, we showed that, when *Magel2* is deleted, *Necdin* expression is increased and vice versa. Such overexpression could induce a compensatory mechanism, but *Necdin* or *Magel2* overexpression could also be responsible for part of the phenotype in *Magel2-*KO or *Necdin-*KO mice, respectively. Indeed, it has been reported that a 2-fold increase in *Necdin* induced ASD-related behaviors (52). Similarly, overexpression of the N-terminal region of Magel2 is lethal at embryonic or neonatal stages, whereas normal production of this Magel2 protein is not lethal, indicating the toxic effects of the overexpression of the protein (53). Thus, the *Del Ndn-Magel2* mouse model with a deletion including both genes is more relevant to study PWS from a genetic and transcriptional point of view since, in this mouse model, both genes are deleted without the overexpression of *Magel2* or *Necdin* that could occur in the single KO mice (i.e., in *Magel2-* or *Necdin-*KO mice).

Our teams have created and extensively studied the *Necdin^tm1.1Mus^*– and *Magel2^tm1.1Mus^–*KO mouse models from which *Del Ndn-Magel2* mice have been created. We and others, such as the R. Wevrick’s team (University of Alberta, Edmonton, Alberta, Canada), have revealed important and specific roles for each of these genes in the pathophysiology of PWS, and these mouse models have been used in several preclinical studies. However, phenotypic differences were observed between the 6 different *Necdin-*KO models and the 3 different *Magel2-*KO models that have been described (Table 1) (11). Those differences might be explained by the genomic constructions causing either a deletion and lack of transcripts or the creation of a truncated or fused protein that might induce an additional phenotype to the loss of function of the normal proteins. Furthermore, at least in *Necdin^tm1.1Mus^* and *Magel2^tm1.1Mus^* KO mice, a stochastic and very low expression of the maternal allele has been detected for *Necdin* (43) and *Magel2* (38), respectively. This weak expression was sufficient to alleviate the phenotypes (43) and explained part of the variability between pups from the same litter. Similar weak expression was detected in patients with PWS and might also explain part of the variability in the phenotype (43).

In the present study, we produced several cohorts of *Del Ndn-Magel2* mice in different animal facilities, all on the same C57BL6/J genetic background, and observed variability in the severity of the phenotype (in particular on the mortality rate at birth) linked to the cohort. We also observed this variability with the *Magel2^tm1.1Mus^* mice. We could correlate the phenotypic variability of *Del Ndn-Magel2* mice with the different levels of sanitary status of the animal facilities in which animals were housed — i.e., in terms of pathogens and sterility of food and water. For example, when animals were housed in a pathogen-free animal facility and were fed with sterile food, the phenotype was much less severe than when they were housed in a conventional animal facility. It is interesting to note that MAGE genes, conserved in all eukaryotes, play a role in adaptation to stress (e.g., nutritional, genotoxic, and heat) (51) and that, in the absence of stress, their function is not revealed. It is therefore likely that the phenotype of *Del Ndn-Magel2* mice could be highly dependent on the environment (including pathogens, food, and water), and this issue should be taken into consideration in all animal houses, where the absence of pathogens does not reflect real-life conditions. The environment is also a challenge in patients with PWS. For example, respiratory failure is the most common cause of death (73% of infants) in PWS and occurs in a stressful environment (e.g., airway infection, hypercapnia, hypoxia). This observation reveals the infants’ inability to adapt their respiratory response (54).

Feeding difficulties, respiratory deficits, and low body weight are among the first symptoms observed in babies with PWS. These early phenotypic traits are also found in *Del Ndn-Magel2* neonates that either died without milk in their stomachs or with cyanotic appearance. The surviving pups displayed a low body weight and poor somatic growth from birth to weaning. The absence of milk in the stomach was also observed in *Magel2^tm1.1Mus^*–KO pups and was shown to result from a suckling deficit caused by a defect in OT maturation and release. A similar reduction in the number of OT neurons was reported in the *Ndn^tm1-Mus^*–KO mice (32). Interestingly, we also found a defect in OT maturation in *Del Ndn-Magel2* pups. The cyanotic appearance was also observed in *Necdin^tm1.1Mus^*–KO neonates. It reflects respiratory distress that was extensively studied in *Necdin^tm1.1Mus^*–KO mice and is characterized by frequent apneas, irregular rhythms in basal conditions, and altered ventilatory response to hypercapnia (33). We did not observe cyanosis in *Del Ndn-Magel2* pups born in a pathogen-free animal facility, but during adult life, we detected differences in ventilatory parameters in normal conditions and also an abnormal persistence of apneas after a hypercapnic challenge, revealing alleviated respiratory deficits.

One of the hallmarks of PWS is the progression of symptoms over time. The motor and possibly sensory deficits underlying hypotonicity of infants with PWS tend to disappear later in childhood. We did not observe motor deficits in *Del Ndn-Magel2* pups, but our results suggest a delay in maturation of touch/proprioception sensory modalities. PWS is also characterized by endocrinopathies including growth deficiency, hypogonadism, and delayed puberty onset, which we also observed in our *Del Ndn-Magel2* mouse model. The feeding difficulties found in infants with PWS are replaced by hyperphagia, leading to the development of obesity (55). Despite the development of many rodent models of PWS, only 2 models have somewhat reproduced a phenotypic trait related to hyperphagia and/or weight gain. A strain of *Necdin-*KO mice (on Imprinting Control Region [ICR] background) (56) become obese on a high-fat diet. *Snord116-*KO mice show growth delay marked by reduced body weight but consume more food in proportion to their body weight, which was interpreted as hyperphagia (13, 14). In addition, a subset of mice in which *Snord116* expression is reduced in part of the adult hypothalamus show hyperphagia and obesity (15). *Magel2-*KO mice do not become obese but tend to gain more weight after weaning (21). All the other mouse models of PWS tend to be leaner than control littermates (11). Similarly, male *Del Ndn-Magel2* mice have reduced body weight and fat mass and have normal food consumption (even when normalized to body weight), energy expenditure, and locomotor activity. In addition, male *Del Ndn-Magel2–*KO mice display an improved glucose tolerance similar to the ameliorated glucose metabolism generally observed in patients with PWS (45). Altogether, these findings suggest that several metabolic and feeding behavior defects observed in PWS might partly be independent of *Magel2* and *Necdin*.

Behavioral changes are present throughout the patient’s life. Anxiety, interactions with novel objects or social interactions are altered in *Necdin*-KO (37) and *Magel2*-KO mice (24, 27), with a variability in the severity of the phenotype, partly due to the environmental context. *Del Ndn-Magel2* mice showed a preference for the novel object versus a familiar one and for a congener mouse versus an empty cage. However, the interest in moving toward the novel object or the time spent to socialize is reduced compared with the controls. Importantly, the cohort of *Del Ndn-Magel2* mice investigated for adult behavior was generated in a “sterile” context (associated with 0% lethality), which could explain why we observed a milder alteration of social behavior compared with *Magel2* mice. Intriguingly, recent studies have implicated alterations in GnRH number and function, including during early postnatal development, in adult social interaction and cognition (47, 57, 58). *Del Ndn-Magel2* mice show a selective reduction in the number of GnRH cell bodies in the olfactory bulb, a neuronal population recently shown to be involved in processing socially relevant odors required for congener recognition (59). This finding provides a potential link between GnRH neuron alterations and social behavior deficits observed in *Del Ndn-Magel2* mice. The control and maintenance of GnRH production in the brain after birth could be an interesting actionable target for managing cognition and social interactions in PWS. Similar approaches have shown promise in other chromosomal disorders such as trisomy 21 (58).

Because *Magel2* and *Necdin* have a circadian expression (36), transcriptomic analyses were performed on hypothalami collected in the middle of the day (ZT6) and in the middle of the night (ZT18) to identify alterations in rhythmic patterns of gene expression. We found that the day/night differential expression of the master core-clock gene *Arntl* (also known as *Bmal1*) was blunted in *Del Ndn-Magel2* mice. While a phase shift in its circadian pattern cannot be excluded, its decreased expression level at night is consistent with the reduction in BMAL1 protein expression observed after *Necdin* repression in U2OS cells (36). It has been previously shown that *Necdin* regulates BMAL1 stability and the amplitude of the rhythm of several core-clock genes such as *Bhlhe40* (Dec1) and *Bhlhe41* (Dec2) (36). Accordingly, some core-clock genes other than *Bmal1*, such as *Npas2*, *Nr1d1* (Rev-erbα), *Nr1d2* (Rev-erbβ), *Bhlhe40*, and *Bhlhe41*, were also affected in *Del Ndn-Magel2* mice. These genes either lost their day/night differential expression (*Bhlhe40*, *Nr1d1*, and Nr1d2), exhibited altered levels at night (*Npas2* and *Bhlhe41*), or similar to *Arntl*, displayed both alterations (*Nr1d1*). We found that genes affected in *Del Ndn-Magel2* mice were significantly overrepresented in the category “rhythmic process” with 21 identified genes (data not shown). In addition to the core-clock genes mentioned above, other genes in the 21-gene list are histone-modifying enzymes such as *Crebbp*, *Ep300*, or *Kmt2a*, known to play a crucial role in circadian-clock-output gene expression by contributing to the rhythmic recruitment of the CLOCK-BMAL1 transcription factor complex to circadian gene promoters (60). Some genes regulated in the *Del Ndn-Magel2* mice could also serve as hubs between core-clock genes and circadian-clock-output genes.

Previous transcriptomic analyses performed on post mortem hypothalamic tissues of patients with PWS identified changes in molecular pathways involved in neuronal loss, neuroplasticity, and neuroinflammation (48). Interestingly, we found 261 genes that were commonly dysregulated in both *Del Ndn-Magel2* mice and patients with PWS, and the functions of these genes appeared to be related to synaptic and axonal function, making the *Del Ndn-Magel2* mice a particularly relevant model from which to study neurodevelopmental and neurocircuits defects associated with PWS. In support of the alteration of genes involved in axonogenesis, we observed structural alterations in POMC neuronal circuits in *Del Ndn-Magel2* mice, similar to what we previously reported in the *Magel2*-KO model (29).

In conclusion, we observed a wide range of phenotypic traits in *Del Ndn-Magel2* mice that cover neurodevelopmental and behavioral symptoms of PWS, recapitulating part of the phenotypes observed in *Necdin-* and *Magel2*-KO mice. It would be important to compare the phenotype of *Magel2* and *Necdin* single KO with that of *Del Ndn-Magel2* mice in the same experimental and environmental conditions to rigorously determine whether the phenotype of *Del Ndn-Magel2* mice results from the addition of the *Magel2* and *Necdin* single KO phenotypes only or whether it results from a more complex phenotype. Nevertheless, the *Del Ndn-Magel2* mice with a deletion including both genes is more relevant from which to study PWS from a genetic and transcriptional point of view since in this mouse model both genes are deleted without the overexpression of *Magel2* or *Necdin* that could occur in the single KO mice for *Magel2-* or *Necdin-*KO mice. Although *Del Ndn-Magel2* mice do not display hyperphagia and obesity as most models of PWS, this mouse model is a pertinent and relevant preclinical model to study many other symptoms observed in PWS.

## Methods

### Sex as a biological variable

Our study examined male and female animals. Both sexes were pooled when no sex differences were found.

### Transallelic recombination to generate the Del Ndn-Magel2–KO mouse model and housing conditions

We used a Cre-loxP site-specific recombination strategy to mediate efficient transallelic recombination between a loxP site in the *Necdin*-KO mouse (*Ndn^tm1-Mus^*) (32) and a loxP site in the *Magel2*-KO mouse (*Magel2^tm1-Mus^*) (20) in a transgenic mouse expressing the *Hprt*-Cre recombinase (61). This approach can be used because both loxP sites associated with the mutated *Necdin* allele or the mutated *Magel2-*KO allele, located on a different chromosome 7, are oriented in the same direction, allowing the creation of 1 allele with the deletion of the DNA region between both loxP sites, deletion of *Necdin,* and deletion of the 5’ coding part of *Magel2*. We used an *Hprt-*Cre driver mouse in which a strong CAG promoter is inserted into the X-linked *Hprt* locus allowing a strong, constitutively expressed, Cre recombinase. *Hprt*-Cre/*Magel2*–KO (–m/+p) male mice were generated and then crossed with heterozygous *Necdin*-KO female mice (–m/+p).

### Behavioral tests in pups

Behavioral tests performed during the 2 first weeks of postnatal life followed a protocol described in ref. 44 and are detailed in Supplemental Methods.

### Behavioral studies in adult mice

Behavioral tests in adulthood were performed by Phenotype Expertise, Inc. with an expert behaviorist. The number of tested animals was based on previous publications and phenotype expertise experience. For all tests, mice were first acclimated to the behavioral room for 30 minutes. All tests are described in Supplemental Methods.

### Metabolic phenotyping

Animals were weighed daily from birth to weaning (P21) and weekly from P21 to P168 using an analytical balance. Body length was measured on anesthetized mice at P24 and P60 with a rigid metric ruler. Body composition analysis was conducted weekly from P25 to P168 using a Minispec LF Series (Bruker Corporation). Fat mass, lean mass, and free body fluid measurements were expressed as a percentage of total body weight. Infrared pictures were taken with a hand-held camera (E8 camera model, with an accuracy of 2% max 2°C, FLIR Systems) on freely moving and unshaven mice at P120 to assess interscapular brown adipose tissue (BAT) temperature. Food intake, O_2_ consumption and CO_2_ production, energy expenditure, RER (i.e., VCO_2_/O_2_), and locomotor activity (*x* and *y* axes) were monitored in fed mice, 24-hour fasted mice, and after a 48-hour refeeding period at P180 using a combined indirect calorimetry system (TSE Pheno Master Systems GmBH). Experimental details on the metabolic phenotyping are described in Supplemental Methods. Glucose tolerance tests were conducted in mice at approximately P130 of age through i.p. injection of glucose (2 g/g body weight) after 6 hours of fasting. Blood glucose levels were measured at 0, 15, 30, 60, 90, 120, and 150 minutes after injection, as previously described (62).

### Reproductive phenotyping

Starting on P21, female mice were inspected daily for imperforation of the vaginal membrane (“vaginal opening,” VO). After that, vaginal smears were collected daily and analyzed under the microscope to identify the onset of puberty (first appearance of 2 consecutive days where vaginal smears contained cornified cells) and the specific day of the estrous cycle, as described previously (63, 64). Male mice were checked daily for balano-preputial separation as an external sign of puberty onset.

### Hormone assays

Serum insulin levels were measured in P130 mice before, 15, and 30 minutes after i.p. glucose administration (2 g glucose/kg body weight) using commercially available insulin ELISA kits (Mercodia). Serum leptin levels were measured in fed mice at P160–P240 using commercially available leptin ELISA kits (DuoSet Ancillary).

### In vivo plethysmography recordings

Unrestrained, nonanesthetized mice were monitored at P30 for their breathing activity using whole-body plethysmography equipment (EMKA Technologies) as described in Supplemental Methods.

### Neuroanatomical studies

Paraformaldehyde-fixed brains were frozen, sectioned at 30 μm thickness, and processed for immunofluorescence using standard procedures (65). Brain sections were counterstained with DAPI to visualize cell nuclei. For the iDISCO+ immunolabeling, whole brains were first dehydrated before being incubated with the primary and secondary antibodies and cleared as previously described (66). The primary antibodies used for IHC were as follows: rabbit anti-POMC (1:10,000, H-029-30, Phoenix Pharmaceuticals), rabbit anti-AgRP (1:1,000, H-003-53, Phoenix Pharmaceuticals), mouse anti–neurophysin 2 clone PS38 (1:1,000, MABN844, Merck Millipore), rabbit anti-GnRH (1:3,000, 26950-1-AP, Proteintech), and rabbit anti-Necdin (1:500, 07-565, Merck Millipore). The primary antibodies were visualized with goat anti–rabbit IgG conjugated with Alexa Fluor 568 (1:500, A11011, Invitrogen), donkey anti–rabbit IgG with Alexa Fluor 568 (1:1,000, A10042, Invitrogen), or anti-mouse IgG conjugated with Alexa Fluor 647 secondary antibodies (1:500, A21235, Invitrogen). Image analysis was then performed as described in Supplemental Methods.

### In situ hybridization

Brain sections from embryos were postfixed with 4% paraformaldehyde and processed for in situ hybridization using antisense digoxigenin-labeled *Necdin* and *Magel2* riboprobes as previously described (12). In situ hybridization on adult brain sections were performed using fluorescence labeling as previously described (43). The *Ndn* riboprobe (290 bp) hybridized to the 3′ UTR of the *Ndn* mRNA (nt 2,130–2,420; accession no. D76440). The *Magel2* riboprobe (318 bp) hybridized to the 3′ part of the Magel2 ORF (nt 4,226-4,544 accession no. NM_013779.2).

### RNA extraction and qPCR analyses

For Figure 1D, WT and mutant newborns were sacrificed at P0. The hypothalamus was quickly dissected on ice and rapidly frozen in liquid nitrogen before being stored at −80°C. Total RNA was isolated using the RNeasy Mini Kit (Qiagen, 74104), according to the manufacturer’s protocol, and cDNAs were obtained by reverse transcription using QuantiTect Reverse Transcription Kit (Qiagen, 205311), starting with 600 ng of total RNA. We performed qPCR using Sybrgreen-based application (on the LightCycler 480 Instrument).

For Figure 2, D and E, *Del Ndn-Magel2* mice were sacrificed at P60, and the hypothalamus was quickly dissected as described above. Total RNA was isolated using E.Z.N.A. Total RNA Kit (Omega BIO-TEK, R6834) according to the manufacturer’s protocol and cDNAs were obtained by reverse transcription using High-Capacity cDNA Reverse Transcription Kit (Applied Biosystem, 4374967), starting with 1 μg of total RNA. We performed qPCR using GoTaq qPCR Master Mix (Promega, A6002) according to the manufacturer’s protocol with QuantStudio 3 Real-Time PCR Systems.

mRNAs and quantity of genes of interest transcripts were normalized with *Gapdh* and *Actin* reference transcripts as previously described (67). The qPCR primers used are listed in Supplemental Table 2.

### RNA expression analysis by RNA-Seq

Mice were housed in a 12-hour light/12-hour dark cycle (LD) and were sacrificed 6 hours after light on (DAY group) or 18 hours after light on (NIGHT group).

#### Total RNA-Seq.

The construction of Illumina DNA libraries was prepared with Illumina Sample Preparation kit with rRNA depletion. Strand-specific RNA-Seq was done on Illumina HiSeq with 2 × 150 bp sequencing configuration (30 million reads per sample on average were obtained).

#### Mapping of total RNA-Seq.

Paired-end reads (150 bp) were aligned to the Mouse reference genome (UCSC mm10) using HISAT2 (68).

#### Quantification of RNA levels for each gene, and differential expression analysis.

FeatureCounts (69) was used to quantify the number of counts for each mRNA. Differential expression analysis was performed using DESeq2 (70). Low-count genes (<10 reads in total) were removed before running DESeq2.

#### GO analysis.

GO analysis was performed using Panther web service (71) (https://pantherdb.org). An enrichment test was performed for BP. The analysis performed was a statistical overrepresentation test. Categories with adjusted *P* values (Benjamini–Hochberg) smaller than 0.05 were reported.

### Statistics

Statistical analyses were conducted using GraphPad Prism (version 10.2.2). To determine statistical significance between 2 independent groups, we used either the parametric Student’s unpaired *t* test (2-tailed) or the nonparametric Mann-Whitney *U* test, depending on the sample size. When evaluating multiple groups, we utilized either a 1-way ANOVA with post hoc Tukey’s multiple comparisons test or a 1-way ANOVA with repeated measures and Šidák multiple-comparison test for pairwise comparisons between the groups. Other statistical tests used are indicated in the figure legends. The χ^2^ test was used to determine whether a categorical variable followed a hypothesized distribution. Statistically significant outliers were identified using Grubb’s test. *P* ≤ 0.05 was considered statistically significant.

### Study approval

All experiments were performed in accordance with the *Guide for the Care and Use of Laboratory Animals* (National Academies Press, 2011) and the European Communities Council Directive of September 22th 2010 (2010/63/EU, 74) and the approved protocol (APAFIS 13387–2017122712209790 for the studies performed in Lille and accreditation no. B13-055-19 for the studies conducted in Marseille) by the Ethical Committee of the French Ministry of Agriculture.

### Data availability

The data reported in this paper have been deposited in the Gene Expression Omnibus (GEO) database (www.ncbi.nlm.nih.gov/geo; accession no. GSE162751) and in the Supporting Data Values file.

## Author contributions

FM, SGB, and VP conceived and designed the project. FS and FM conceived the mouse model. PYB, AS, FS, JB, CM, DB, FO, CS, FM, AMFB, JK, and EC performed experiments. FM, SGB, VP, PYB, AS, FS, JB, CM, DB, MSA, FO, CS, FM, AMFB, JK, and EC analyzed data. PYB, AS, AMFB, FM, and SGB wrote the manuscript. All the authors read and approved the manuscript. FW performed experiments.

## Supplementary Material

Supplemental data

Supporting data values

## Figures and Tables

**Table 1:**
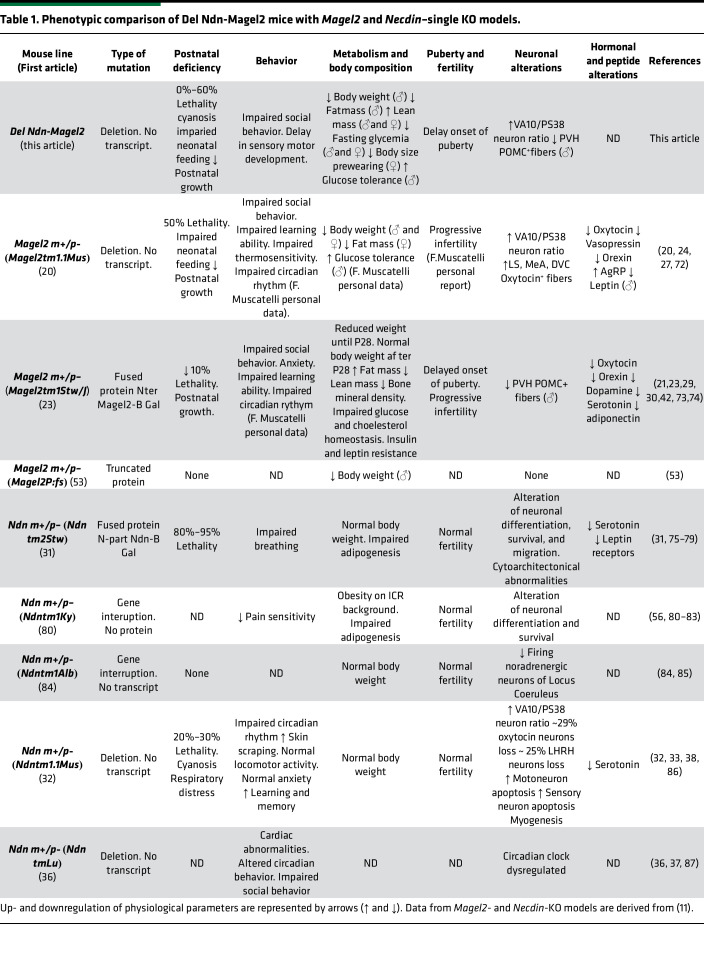
Phenotypic comparison of Del Ndn-Magel2 mice with *Magel2* and *Necdin*–single KO models.

## Version 1 03/06/2025

In-Press Preview

## Version 2 04/22/2025

Electronic publication
