# Supplementary material for: Investigation of a mouse model of Prader-Willi Syndrome with combined disruption of Necdin and Magel2
Source: JCI Insight. 2025 Mar 6;10(8):e185159. doi: 10.1172/jci.insight.185159 (PMC12016932; doi:10.1172/jci.insight.185159)
Supplement: Supplemental data [file jciinsight-10-185159-s245.pdf]

Barelle *et al.*

Supplemental Methods, Results, Tables and Figures

Supplemental Methods

Behavioral tests in pups

The age of eye and auditory canal opening was checked by visual inspection. The righting response was performed by scoring the time for righting, *i.e.*, the ability of the pup put on its back to stand up on its paws (scoring the time for righting). The rooting response assessed the disappearance of the rooting reflex triggered by bilateral stimulation of the snout. 3 consecutive tests were performed and scored, with the total score plotted on a graph (Score 0: pup roots; score 1: pup stops to root; score 2: pup removes the head from the fingers). The paw position test is indicative of the appearance of an adult pattern when all four paws were placed flat on the ground (score 1 per paw placed flat on the ground). The bar holding test consisted in scoring the time during which the pup is able to hang when putting its ventral face on the bar just at the level of the diaphragm, the position was unstable and the pup hanged onto the bar to prevent falling. The pulling up on the bar after hanging test consisted in performing the bar holding test and assessing the pulling up on the bar after hanging. Three scores were given: score 1: the pup pulled up by drawing the bar towards him; score 2: the pup braced himself with his hind feet; score 3: the pup stood or walked on the bar. The climb the slope test consisted of the pup climbing up a 30° slope. The pup is positioned with the head downward (stay still), it turned the head, then the body at 90°, then at 180° and climbed the slope. The response is due to the maturation of semicircular canals (inner ears). In the vertical climbing test, the pup climbed along the wire mesh of vertical grid. The score was the number of mesh wires that the pup covers in 20 sec. In the cliff drop avoidance test, the pup should turned away from the edge of a step, starting with the forepaws dangling over the edge of the slice (score 0: it remained motionless or fell from the edge; score 1: it turned the head aside; score 2: it turned the head aside, pulled back the forepaws from the cliff and put them on the slice; score 3: it initiated a backward movement.

Behavioral studies in adult mice

Rotarod. Mice were challenged to walk on a rotating rod that increases in speed over a predetermined period of time (for 5 min) until the animal falls. The latency to fall from the rod was determined and taken as a measure of motor function. The test was performed over three successive days. The daily trial consisted of three measures spaced by 5 minutes, a mean of the three measures was considered for each day.

Elevated plus maze. The device consists of a labyrinth of 4 arms of 5 cm width located 80 cm above the ground. Two opposite arms are open (without wall) while the other two arms are closed by side walls. The light intensity was adjusted to 20 Lux on the open arms. Mice were initially placed on the central platform and left free to explore the cross-shaped labyrinth for 5 minutes. The maze was cleaned and wiped with H_2O and with 70% ethanol between each mouse. Animal movement was video-tracked using Ethovision software 11.5 (Noldus). Time spent in open and closed arms, the number of entries in open arms, as well as the distance covered, are directly measured by the software.

Open field. The open field test was performed in a 40 x 40 cm square arena with an indirect illumination of 60 lux. Mouse movement was video-tracked using Ethovision software 11.5 (Noldus) for 10 minutes. Total distance traveled and time in center (exclusion of a 5 cm border arena) are directly measured by the software. Grooming (time and events) and rearing were manually counted in live using manual functions of the software, by an experimented behaviorist. The open-field arena was cleaned and wiped with H_2O and with 70% ethanol between each mouse.

Spontaneous Social Interaction in an open field arena. Animals were first habituated to the apparatus for 30 min. Social interaction was measured using pairs of mice from different housing cages, and having the same genotype, the same gender, and approximately the same body weight. Each pair was placed in the open field arena for 15 min during which different behavioral parameters reflecting social interaction between the two mice were recorded (following and sniffing). A social interaction index was defined as the percentage of counts and duration of social events over the total social and individual events.

New object recognition. The arena used for the novel object recognition test was the same used for the open-field test. The arena was cleaned and wiped with 70% ethanol between each mouse. Two identical objects (50 ml orange corning tube) were placed in the opposite corners of the arena, 10 cm from the side walls. The tested mouse was placed at the opposite side of the arena and allowed to explore the arena for 10 min. After 1h, one object was randomly replaced with another novel object, which was of similar size but differ in the shape and color with the previous object (white and blue lego bricks), the other object (same object) was kept. Then, the same mouse was placed in the arena and allowed to explore the two objects (a new and an "old" familiar object) for 10 min. The movement of the mice was video-tracked with Ethovision 11.5 software. Time of exploration of both objects (nose located in a 2 cm area around object) was automatically measured by the software. The traveled distance to reach the novel object or the same object (old object) was measured.

Three-chamber social preference test. The three-chamber apparatus consisted of a Plexiglas box (50x25 cm) with removable floor and partitions dividing the box into three chambers with 5-cm openings between chambers. The task was carried out in four trials. The three-chambers apparatus was cleaned and wiped with 70% ethanol between each trial and each three-chamber test experiments. In the first trial (habituation), a test mouse was placed in the center of the three-chamber unit, where two empty wire cages were placed in the left and right chambers to habituate the test mouse to arena. The mouse was allowed to freely explore each chamber. The mouse was video-tracked for 5 min using Ethovision software. At the end of the trial, the animal was gently directed to the central chamber with doors closed. In the second trial (social exploration), an 8 weeks old C57BL/6J congener mouse (S1) was placed randomly in one of the two wire cages to avoid a place preference. The second wire cage remained empty (E). Then, doors between chambers were opened and the test mouse was allowed to freely explore the arena for 10 min. The measure of the real social contact is represented by the time spent in nose-to-nose interactions with the mouse. This test was performed using grouped-house mice.

Metabolic phenotyping

The mice were acclimated to the monitoring chambers for two days prior to the start of the experimental protocol. Metabolic data were collected over six consecutive days. The first three days (D1-3) were used to assess basal metabolism under standard *ad libitum* feeding condition. On the fourth day (D4), Food pellets were removed at 9:00 AM to initiate a 24-hour fasting period, during which metabolism under fasting conditions was assessed. Food pellets were reintroduced at 9:00 AM on the fifth day (D5) and metabolism during *ad libitum* refeeding was monitored for 48 hours, from D5 to D6. These physiological measures were performed at the Mouse metabolic phenotypic platform of the University of Lille.

In vivo plethysmography recordings

A constant air flow (0.5 l/min) circulated inside the 200 ml plethysmography chambers (maintained at 25 ± 0.5℃) using a vacuum pump (Vent 4, EMKA, Paris, France). Calibration of the amplitude of the signal was ensured by injecting 1 ml of air into the chamber during each recording session. Analog signals were acquired through a usbAMP device and processed using EMKA technologies IOX software (EMKA Technologies, Paris, France). Respiratory parameters (frequency, tidal volume, minute ventilation, apneas and irregularity score) were analysed using the Spike2 software (Cambridge Electrical Design, Cambridge, UK). Apneas were defined as a prolonged expiratory time equivalent to the loss of two respiratory cycles. The day before the experiment, wild-type and *Del Ndn-Magel2* littermates were habituated in the plethysmography chambers for 2 hours to reduce stress effect on breathing. Respiratory responses to hypercapnia were then evaluated by recording breathing activity for 1 hour under normocapnia, followed by a 10-minute hypercapnic challenge (4% CO2), and a subsequent 30-minute recovery period under normocapnia. Measurements were performed during quiet breathing pre- and post-challenge, and during the last 5 minutes of the challenge. Apneas were also quantified on a minute-by-minute basis during the post-challenge period.

Image analysis

2D images were acquired using a Leica Stellaris 5 confocal Microscope equipped with a 20x objective. Quantifications were performed in two sections per animal at the level of the PVH (Bregma -0.83 to -0.95) and DMH (Bregma -1.79 to -1.91). Slides were numerically coded to obscure the experimental group. The image analysis was performed using the Fiji software (NIH) as previously described (67). For the quantitative analysis of fiber density (for POMC and AgRP), a maximum intensity projection was performed on 5 µm of the Z-stack. The threshold was set manually to ensure that only a positive signal was measured. Images were then binarized, and a standardized region of interest (ROI) was placed within the nucleus of interest. The software then calculated the number of pixels in the ROI corresponding to the signal of interest. This pixel count was finally normalized to the dimensions of the ROI, ensuring comparability across all images. The integrated intensity, which reflects the total number of pixels in the binarized image, was then calculated within the ROI of each image of the stack (58, 59). For the quantitative analysis of cell numbers, PS38-immunopositive cells were manually counted using the Fiji software. Only cells with corresponding DAPI-stained nuclei were included in our counts.

3D imaging was performed on a light sheet Ultramicroscope I (LaVision BioTec) equipped with a 1.1X/0.1NA objective and an Andor Neo 5.5 sCMOS camera. ImspectorPro software (LaVision BioTec) was used for image acquisition, and the z-step between each image was fixed at 5 µm. Image stacks were converted to Imaris files (.ims) using ImarisFileConverter, and 3D reconstruction was performed using "volume rendering" of Imaris 9.8 (Oxford Instruments). Automatic spot detector function of Imaris software was used to count the number of GnRH immunoreactive cell bodies. Anatomic 3D neurons' distribution was done manually.

Supplemental Results

Behavioral studies in Adult mice

We did not observe significant differences between both genotypes in the rotarod test that measures motor function and coordination (Supplemental Figure 4A) and in the open field (OF) test, assessing the spontaneous locomotor activity in a novel environment (grooming, rearing, traveled distance) and giving information about anxiety-related behavior (the time spent in the center zone *versus* border of the arena) (Supplemental Figure 4B). Anxiety behavior was not affected in the mutant mice as shown with the elevated plus maze test that measured the number of entries and time spent in open arms (Supplemental Figure 4D). In addition, we did not observe differences in the spontaneous social interactions between two mice moving freely in the arena of the OF by measuring the frequency, latency to the first interaction, and duration of interaction (Supplemental Figure 4C).

Supplemental Table 1. Commonly dysregulated genes in both *Del Ndn-Magel2* KO mice and PWS patients

DAY TF in *Del Ndn-Magel2* mice (27 items)	Night TF in *Del Ndn-Magel2* mice (69 items)	TF common in DEGs in PWS (Cell Reports) and Day TF in *Del Ndn-Magel2* mice (7 items)	TF common in DEGs in PWS (Cell Reports) and Night TF in *Del Ndn-Magel2* mice (15 items)
AHDC1	AEBP1	Egr3	Aebp1
CIC	ALX3	Fosl2	Alx3
EGR3	ARID5B	Gli1	Arid5b
FOSB	ARNT	Hif3a	Etv6
FOSL2	ARNTL	Prr12	Jund
FOXB1	ARX	Rfx5	Klf8
FOXK1	ATF1	Sox9	Klf9
GLI1	BAZ2B		Npas2
HES5	BHLHE41		Pbx1
HEYL	BNC2		Prox2
HIF3A	EBF1		Rfx4
HLF	ESRRA		Scml4
KMT2B	ESRRG		Smad3
MEF2D	ETV6		Tbx15
NFIX	FLI1		Zbtb20
NR2F6	FLYWCH1		
OTP	FOXP1		
PRR12	GTF3A		
RAX	HDX		
RFX5	IRF2		
SALL1	JAZF1		
SIX3	JUND		
SOX9	KLF12		
SREBF1	KLF8		
UNCX	KLF9		
XBP1	LCORL		
ZKSCAN2	LIN28B		
	MAF		
	MBD2		
	MBD3		
	MEF2C		
	NFIA		
	NPAS2		
	NPAS3		
	NR1D1		
	NR3C2		
	NR6A1		
	NRF1		
	PBX1		

DAY TF in *Del Ndn-Magel2* mice (27 items)	Night TF in *Del Ndn-Magel2* mice (69 items)	TF common in DEGs in PWS (Cell Report) and Day TF in *Del Ndn-Magel2* mice (27 items)	TF common in DEGs in PWS (Cell Report) and Night TF in *Del Ndn-Magel2* mice (69 items)
	PRDM5		
	PRDM6		
	PRMT3		
	PROX2		
	PRRX2		
	RBPJ		
	RFX4		
	RREB1		
	SCMH1		
	SCML4		
	SETBP1		
	SMAD3		
	SMYD3		
	SOX5		
	SP8		
	TBX15		
	TCF7L1		
	TEAD1		
	TERF1		
	TET2		
	THRB		
	TSHZ2		
	ZBTB20		
	ZBTB7C		
	ZEB1		
	ZFHX3		
	ZFPM2		
	ZMAT1		
	ZMAT4		
	ZUP1		

Supplemental Table 2. Primers used for RT-qPCR analyses

qPCR primers		
**Name**	**Forward**	**Reverse**
Necdin	AACAACCGTATGCCCATGA	CTTCACATAGATGAGGCTCAGGAT
Magel2	CTGGGAGATTCAGAGGGCTA	TGCGGAGTGTAGAGGGATTC
Mkrn3	GCC ATT GCG GCA AGA CTA	TTC TTC TCA AGT GTA AGC GAT CC
Snrpn/Snurf	GGG CCC ACC TCC TAA AGA TA	CCT CTG CCA GCT GCT CTT
Snord116	TGA TTC CCA GTC AAA CAT TCC T	ACC TCA GTT CCG ATG AGA GT
Snord115	GGT CAA TGA TGA CAA CCC AAT G	GCC TCA GCG TAA TCC TAT TGA
Actin β	TGACGTTGACATCCGTAAAG	GAGGAGCAATGATCTTGATCT
Gapdh	TGACCTCAACTACATGGTCTACA	CTTCCCATTCTCGGCCTTG

A Spatial mapping of the populations of cells expressing Necdin or Magel2
(http://mousebrain.org/development/)

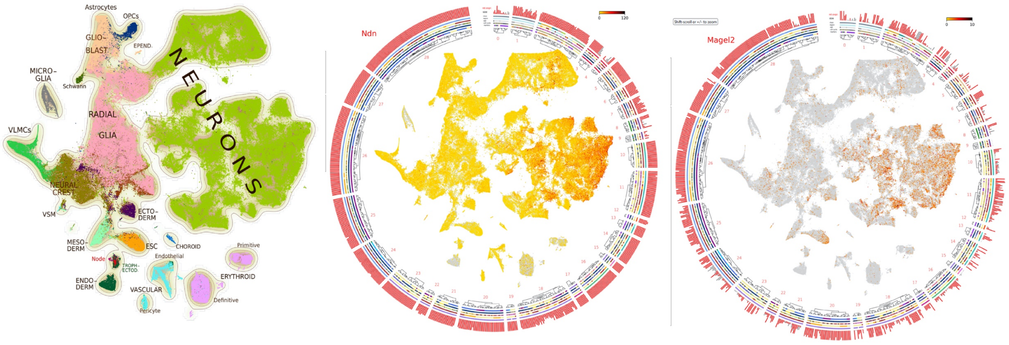

B Relative levels of Necdin and Magel2 in various brain regions and neuronal systems
(http://mousebrain.org/adolescent/genesearch.html)

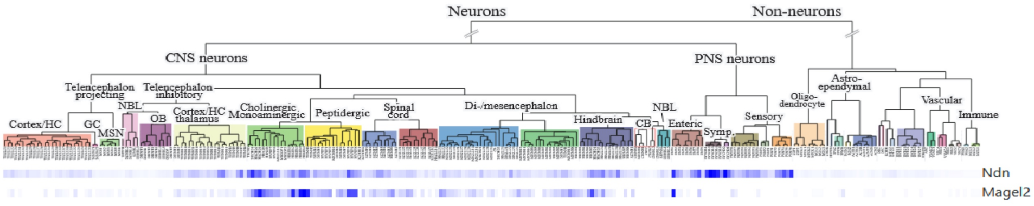

Name	Description	Ndn	Magel2
TEINH8	Interneuron-selective interneurons, hippocampus	3.75	0.00
TEINH7	Interneuron-selective interneurons, hippocampus	6.65	0.00
TEINH6	Interneuron-selective interneurons, cortex/hippocampus	1.38	0.0346
TECHO	Cholinergic interneurons, telencephalon	6.39	0.0580
DECHO1	Cholinergic neurons, septal nucleus, Meissnert and diagonal band	10.0	0.150
HBCHO4	Afferent nuclei of cranial nerves III-V	6.40	0.251
HBCHO3	Afferent nuclei of cranial nerves VI-XII	7.15	0.362
HBADR	Adrenergic cell groups of the medulla	4.02	0.226
HBNOR	Noradrenergic neurons of the medulla	2.04	0.174
HYPEP7	Pmch neurons, hypothalamus	9.07	0.154
HYPEP5	Vasopressin-producing cells, hypothalamus	8.11	0.278
HYPEP4	Oxytocin-producing cells, hypothalamus	5.37	0.266
HYPEP8	Peptidergic neurons, hypothalamus	4.20	0.267

Supplemental Figure 1. Identification of cell types and regions expressing *Necdin* and *Magel2* in the developing mouse brain using scRNA-seq data (from Linnarson lab). (**A**) Wheel plots (tSNE visualization) showing the cell populations expressing *Necdin* and *Magel2* in the brain. (**B**) Dot plots showing relative levels of *Necdin* and *Magel2* in various brain regions and neuronal systems. Data derived from http://mousebrain.org/development/

A **Tests measuring Preweaning Sensorial and Motor Development**
Adapted from L. Roubertoux et al. (2018)

	P 1	P 2	P 3	P 4	P 5	P 6	P 7	P 8	P 9	P 10	P 11	P 12	P 13	P 14	P 15
**Righting response**			X	X	X	X	X	X	X						
**Cliff avoidance**				X	X	X	X	X							
**Adult paw position**					X	X	X	X	X	X					
**Adult walking pattern**						X	X	X	X	X					
**Reaction to slope**						X	X	X	X	X					
**Rooting reflex**							X	X	X	X	X	X	X	X	
**Vertical climbing**									X	X	X	X	X	X	
**Climb the slope**						X	X	X	X	X					
**Bar holding**										X	X	X	X	X	
**Eyelid opening**														X	X
**Opening auditory canal**														X	X

B **Tests measuring behavior at adulthood**

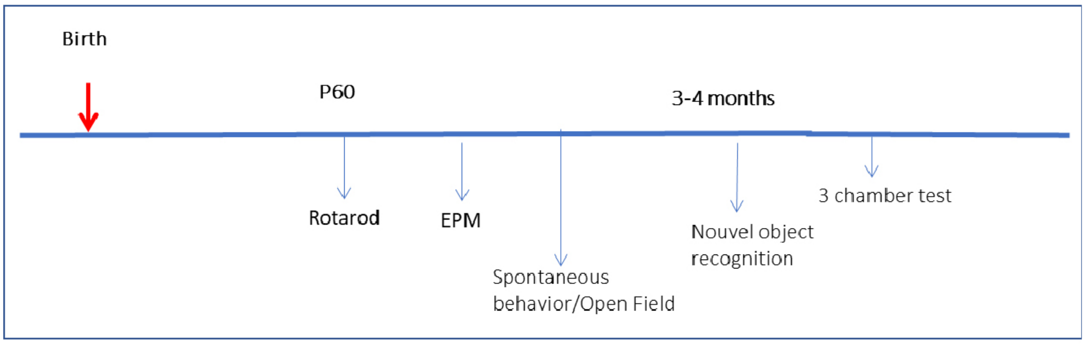

Supplemental Figure 2. Behavioral study paradigm. (**A**) Table listing the eleven tests performed to evaluate preweaning sensorial and motor development in *Del Ndn-Magel2* and WT pups between postnatal day (P) 3 and P15. (**B**) Timeline of behavioral tests perform in adult *Del Ndn-Magel2* and WT mice. *P<0.05, **P<0.01.

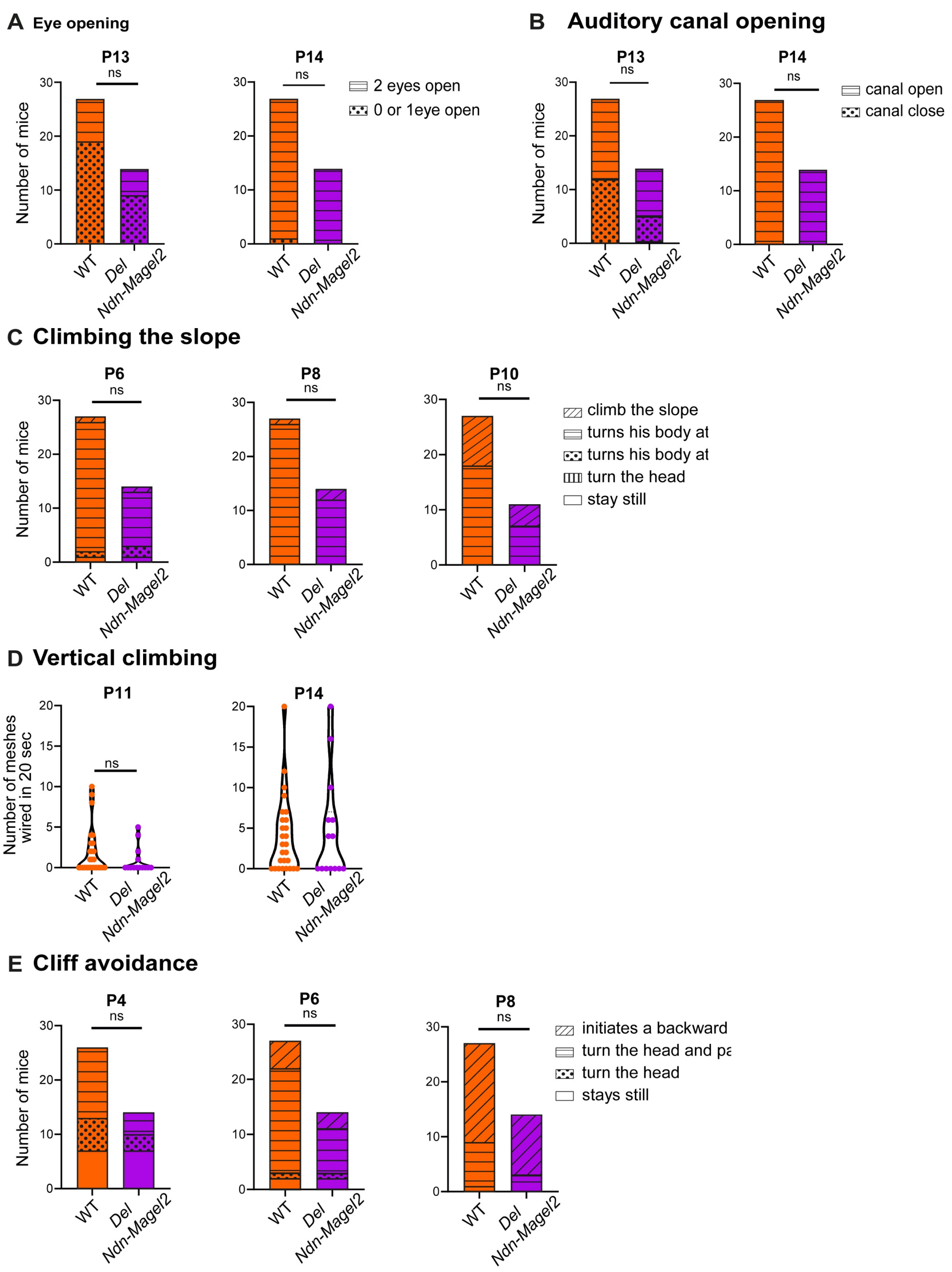

Supplemental Figure 3. Behavioral tests performed in the two first weeks of postnatal life in *Del Ndn-Magel2* KO and WT mice. (**A**) Number of pups displaying eye opening and (**B**) auditory canal opening at P13 and P14 (n = 14-27 animals per group). (**C**) Climbing 30° slope test in pups at P6, P8, and P10 and (**D**) vertical climbing test in pups at P11 and P14 (n = 14-27 animals per group). (**E**) Cliff avoidance test in pups at P4, P6, and P8. Statistical significance between groups was determined by a Chi^2 test (A,B,C,E), or a Mann-Whitney test (D)

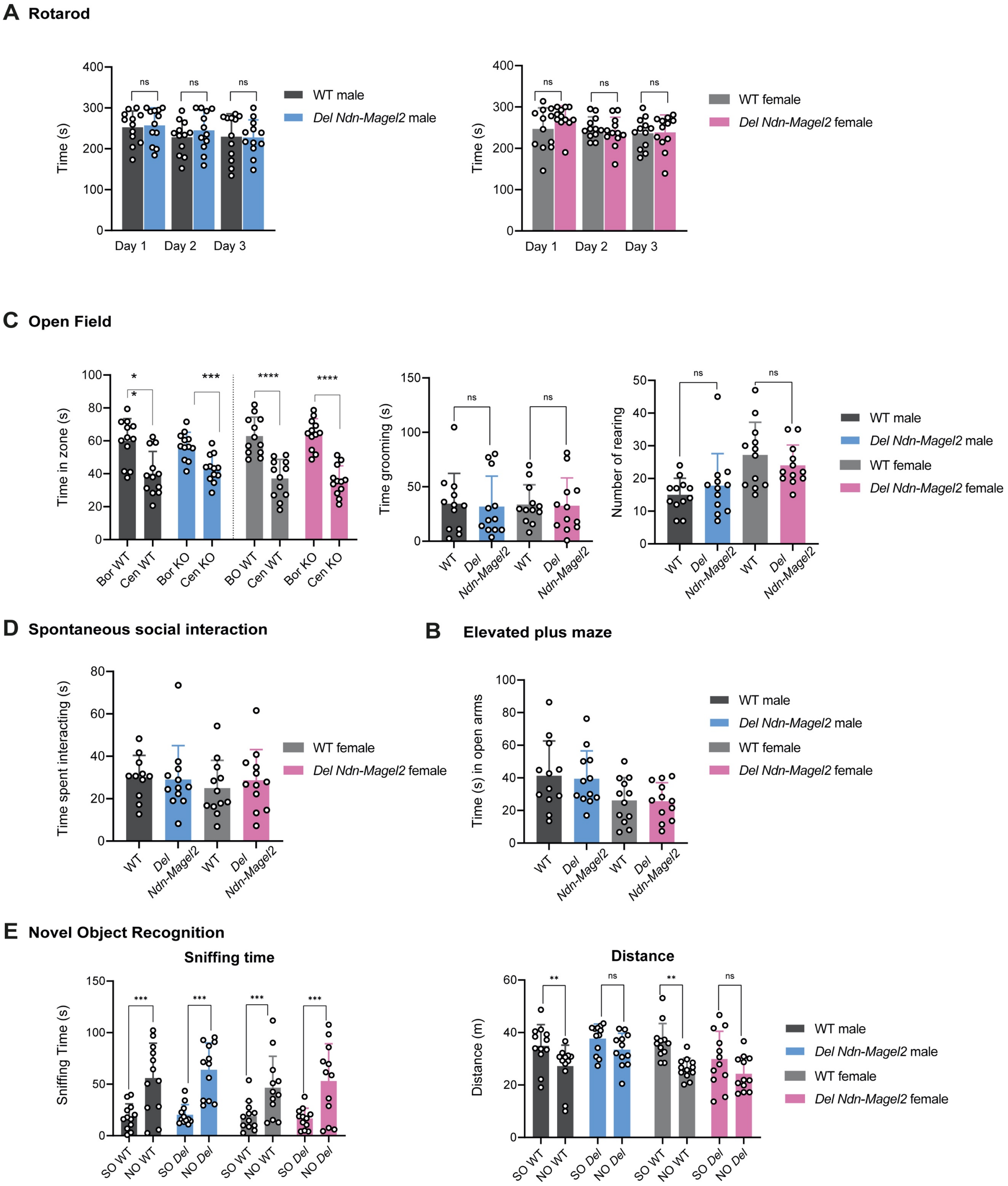

Supplemental Figure 4. Motor ability and spontaneous, anxiety and social behaviors in adult *Del Ndn-Magel2* and WT mice. (**A**) Rotarod, (**B**) open field, (**C**) spontaneous interaction, (**D**) elevated plus maze, and (**E**) novel object recognition tests in adult WT and *Del Ndn-Magel2* mice (n = 12 animals per group). Data are presented as mean ± SEM. Statistical significance between groups was determined by a two-way repeated measures ANOVA (A), a Mann-Whitney test (B,C,D), or a Wilcoxon matched pairs test (E). *$P < 0.05$, **$P < 0.01$.

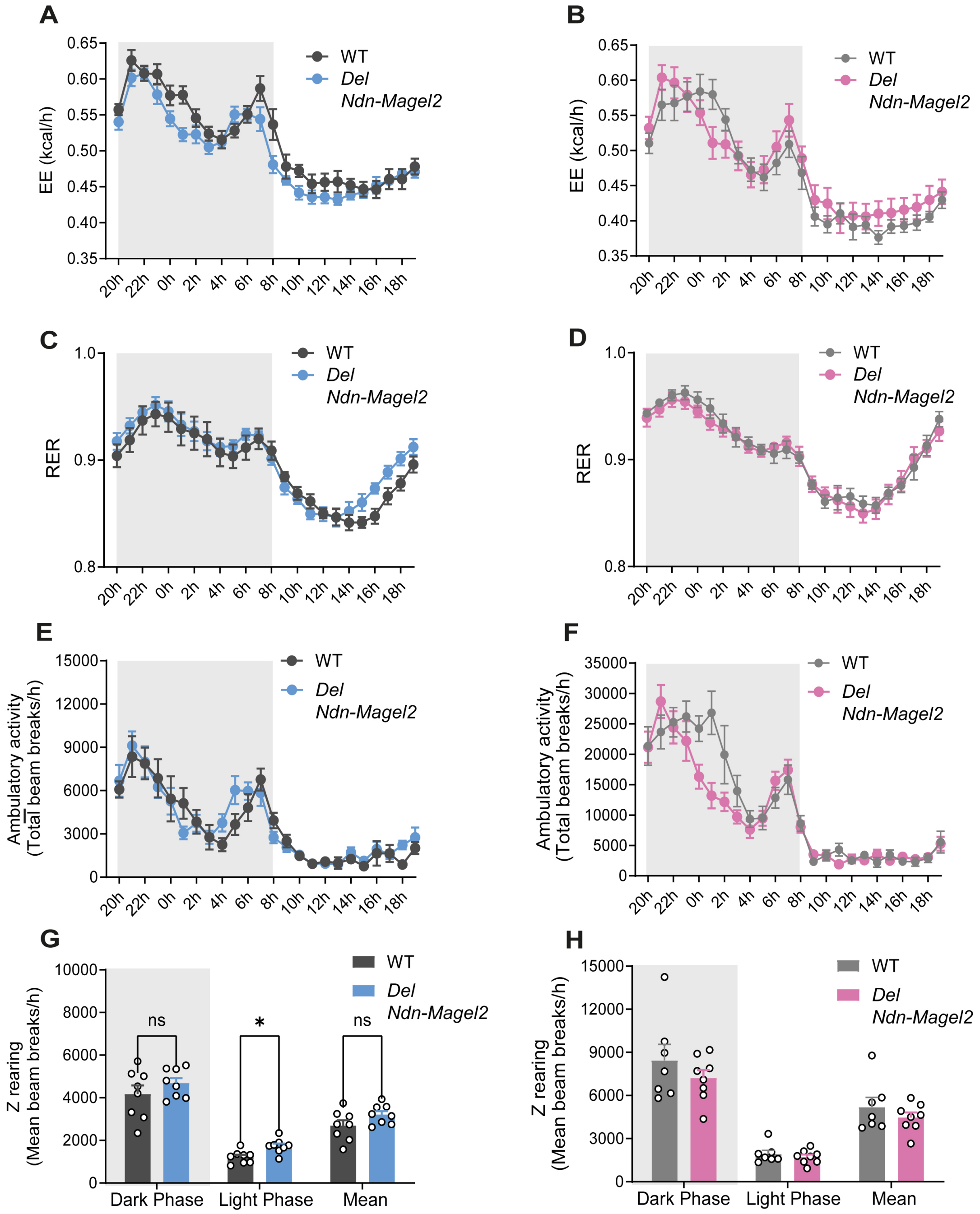

Supplemental Figure 5. *Del Ndn-Magel2* KO mice display normal regulation of energy balance. (**A, B**) energy expenditure, (**C, D**) respiratory exchange ratio (RER), (**E, F**) spontaneous locomotor activity (xy), and (**G, H**) number of z-rearing in (**A, C, E, G**) male and (**B, D, F, H**) female *Del Ndn-Magel2* and wild-type mice at P180 (n = 7-8 animals per group). Data are presented as mean ± SEM. Statistical significance between groups was determined by a 2-way ANOVA followed by an uncorrected Fisher's least significant difference (LSD) test (A-F) or a Mann-Whitney test (G,H). *$P \leq 0.05$.

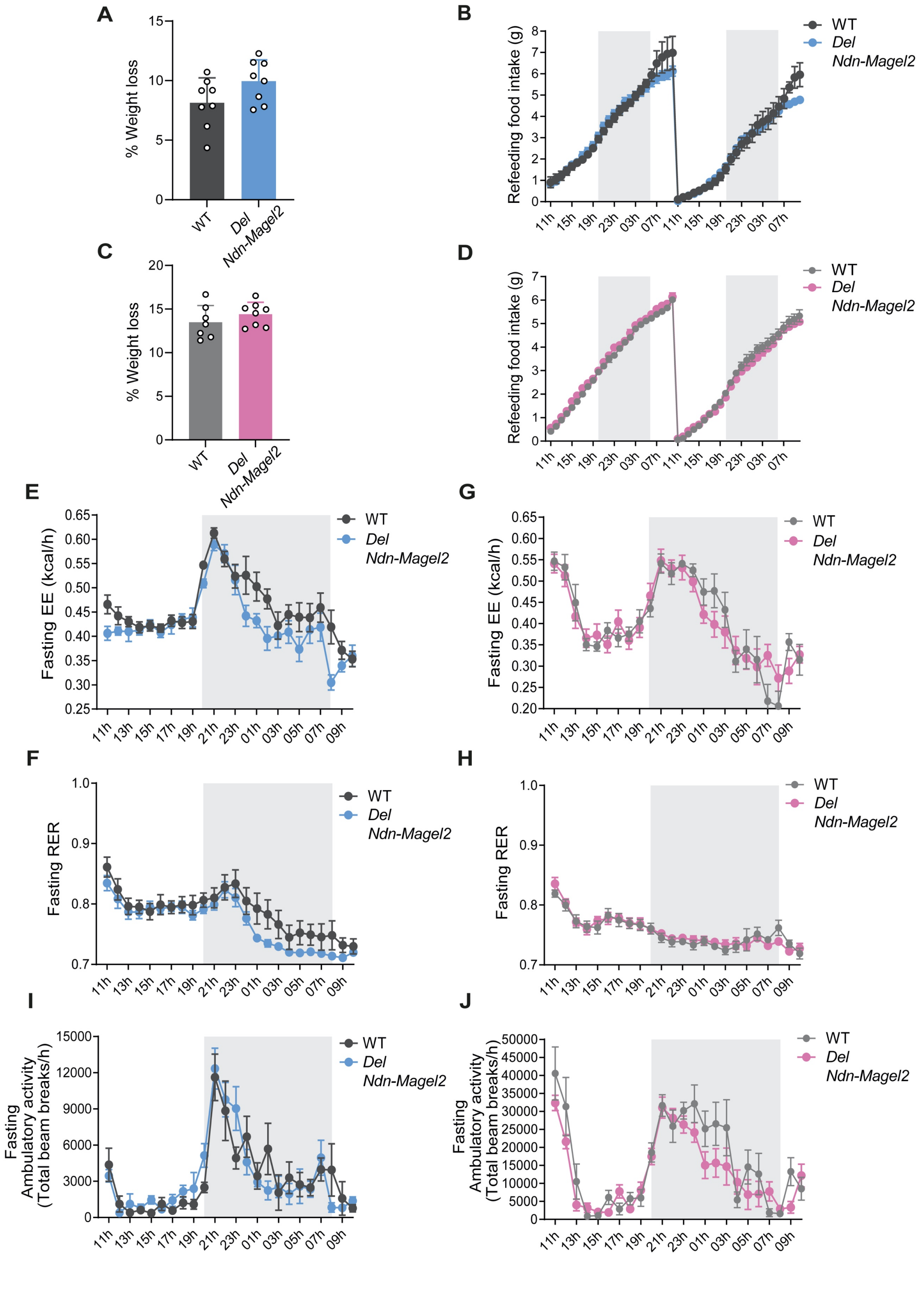

Supplemental Figure 6. Normal response to fasting and refeeding in *Del Ndn-Magel2* mice. (**A, C**) % weight loss after fasting, (**B, D**) cumulative food intake after refeeding, (**E, G**) energy expenditure during fasting, **(F, H)** respiratory exchange ratio (RER) during fasting, (**I, J**) spontaneous locomotor activity (xy), in (**A, B, E, F, I**) male and (**C, D, G, H, J**) female *Del Ndn-Magel2* and wild-type mice at P180 (n = 7-8 animals per group). Data are presented as mean ± SEM. Statistical significance between groups was determined by a Mann-Whitney test (A,B), a 2-way ANOVA followed by an uncorrected Fisher's least significant difference (LSD) test (B-J).
